# Cytotoxic Organometallic Iridium(III) Complexes

**DOI:** 10.3390/molecules30040801

**Published:** 2025-02-09

**Authors:** Irena Kostova

**Affiliations:** Department of Chemistry, Faculty of Pharmacy, Medical University-Sofia, 1000 Sofia, Bulgaria; i.kostova@pharmfac.mu-sofia.bg

**Keywords:** chloridoiridium(III), complexes, anticancer, cytotoxic, review

## Abstract

Iridium complexes attract a lot of attention as highly promising antitumor agents due to their various structures, which offer the modification of their physicochemical and biological effects. Compared to conventional platinum-based drugs, iridium complexes are commonly thought to be more active in tumors, resistant to platinum agents and more stable in air and moisture conditions. Chloridoiridium complexes offer a range of advantages facilitating their rational design, reactivity and photochemical activity, leading to different cytotoxic profiles, diverse mechanisms of action and specific intracellular organelles as targets. They are also known as good light-mediated chemotherapeutics, serving as bioimaging and biosensing agents. The potential biological and photophysical properties of chloridoiridium(III) complexes can be readily controlled by suitable ligand modifications and substitution patterns, providing a wide range of versatile structures. Over the years, numerous different structural types of chloridoiridium complexes have been developed and studied for their antineoplastic activity. In this review, the recent advances in the cytotoxicity studies of chloridoiridium(III) compounds have been summarized. The studied complexes have been categorized in this review according to the number of coordinated ligands, the type of donor atoms, nuclearity of the complexes, etc., allowing for a thorough discussion of the structure–activity relationship.

## 1. Introduction

Iridium belongs to the platinum-group metals, which are the best candidates for the discovery of anticancer drugs [1]. Under the general name of platinum-group metals, the d-elements of the VIIIB group, which are not included in the iron family, are united: ruthenium (Ru) [2], rhodium (Rh), palladium (Pd) [3], osmium (Os), iridium (Ir) and platinum (Pt) [4,5]. These metals have very similar atomic properties and joint distribution in nature. In the compounds of platinum metals, different oxidation states are possible. In the platinum group, the chemical properties of rhodium, iridium, palladium and platinum are much closer to each other than to those of ruthenium and osmium. Rhodium and iridium easily form complexes with an oxidation state of +3, which are kinetically inert.

A particularly important role in recent years has been acquired by the complexes of platinum-group metals used in anticancer treatment. To overcome the side effects and resistance problems of cisplatin and other Pt-based drugs, scientists have tried to design new generation metal antineoplastic complexes [1,2,3,4]. Numerous complexes of platinum and other metals of the group have been obtained and studied for cytotoxic activity, but, only in isolated cases, a noticeable antitumor activity has been observed. Although platinum complexes are recognized as the best carcinostatics, new metal-based antineoplastic drugs have also been developed with proven lower toxicity to treat platinum-resistant tumors. The recent developments in medicinal chemistry have led to the discovery of various other metal-based scaffolds and have prompted further exploration of other transition metal ions, including Ru(III), Pd(II), Rh(IIII), Ir(III) complexes, with promising anticancer activity. Iridium complexes are currently objects of great attention in medicinal chemistry, as antitumor agents with selective physicochemical and biological effects and low systemic toxicity. Additionally, iridium is unique amongst the platinum-group metals because of its broad functional-group tolerance, kinetical inertness and aqueous stability.

Iridium is one of the rarest elements in nature with no identified environmental impact and biological functions. The metal itself is low in toxicity and does not generally cause complications, but iridium compounds should be regarded as extremely toxic [5]. Iridium has the ability to exhibit variable oxidation states ranging from −3 to +9, and the most stable in its coordination compounds are +1 and +3, where +3 is more stable than the +1 oxidation state [6]. As a third-row low-spin d^6^ metal ion, the iridium(III) cation has tunable chemical reactivity and kinetical stability in biological systems. The common coordination numbers in iridium complexes are 4 and 6. The stable nature of Ir(III) ions is an appropriate characteristic for the formation of organoiridium coordination complexes with many bioactive ligands. Iridium complexes have been usually compared with other Pt-group metal complexes, particularly with ruthenium analog compounds. Significant differences in the stability of the complexes depending on the metallic center have been established. For instance, Ru(η^6^-p-cymene) fragments are known to drop the arene ligands, whereas the Ir(η^5^-CpR) fragment is commonly stable under physiological conditions in vivo and in vitro. Nevertheless, in many cases, the final accumulation of toxic effects via the parallel activation of different pathways has resulted in better cytotoxicity exhibited by Ir(III) complexes when compared with Ru(II) complexes [7,8]. The Ir(III) cation has preference to various donor atoms, although nitrogen is the most common. Owing to the relativistic destabilization of its 5d shell, the third-row Ir(III) ion displays a pronounced softness and a subsequent binding preference for ligands holding soft donor atoms such as S atoms or borderline donor atoms such as the endocyclic amino N atoms of nucleobases. This means that analogous Ir(III) compounds should exhibit not only a graded kinetic response but may possibly preferentially bind to different target molecules in biological systems. Their binding preferences can also be further modified by the individual HSAB properties of their facial or meridional co-ligands. The second oxidation state of iridium is Ir(I) with *d*^8^ electron configuration, which is observed in square planar arrangements with most of the donor atoms. Iridium coordination compounds in +1 and other oxidation states are commonly very rare [6].

The success of platinum-based complexes as anticancer agents has led to wide interest in the design of novel metal-based cytostatic drugs. In the early stages of research for new candidates, iridium(III) complexes received less consideration because of their prevailing chemical inertness, which was supposed to account for a lack of cytotoxicity. In fact, iridium(III) complexes, commonly highly water soluble and relatively accessible, have diverse and specific mechanisms of action, including catalytic interference with cellular redox balance, interaction with protein kinases, regulations of non-apoptotic pathways and promising in vitro and in vivo antiproliferative activity [7,8,9,10,11]. Some Ir(III) complexes have exhibited a remarkable ability to interact with DNA, others were able to generate reactive oxygen species (ROS), which allows them to induce cell apoptosis by acting on mitochondria along with DNA [9,10]. Additionally, iridium(III) complexes are good bioimaging and biosensing candidates due to their photo-stability and cell permeability [11]. Their low toxicity, low reduction potential, water tolerance, and outstanding photophysical properties (high quantum yields, long emission lifetimes, large stoke shifts, photon absorption, etc.) make them perfect highly promising anticancer compounds. Their ability to form a wide range of multipurpose structures and ligand substitution patterns offers the possibility to find out specific mechanisms of action and to identify novel targets for this interesting class of compounds [9,10,11].

The different modes of action, reported for a number of the discussed chloroiridium(III) complexes, mean that these compounds can target different subcellular targets and can induce diverse processes in treated tumor cells, compared to other metal-based drugs, involving apoptosis induction on tumor cells, participation in redox reactions, the inhibition of protein activities and protein–protein interactions [12]. Many reports have been devoted exclusively to the comprehensive studies of mechanisms of action, providing an overview of the bioeffect of chloroiridium(III) anticancer complexes as well as the mechanism through which cell death is induced in order to shed light on the structure–activity relationships of newly designed complexes [13]. This mechanism is generally different from that of platinum drugs. It has been reported that chloroiridium(III) complexes predominantly target different intracellular targets (mitochondria, lysosomes, etc.) than DNA, and they commonly induce ROS generation and apoptosis in the tested tumor cells. The redox-mediated mechanism, typical for Ir(III) complexes, refers to the way that a metal-based drug exerts its effect via the disturbance of cellular oxidation–reduction homeostasis. It is directly associated with the electron transfer between the eventual drugs and biological macromolecules, playing a crucial role in numerous cellular bioprocesses, such as antioxidant protection, signaling pathways, enzymatic activity, gene expression and so on). It is a fundamental pharmacological concept, and its consideration is critical for drug development. Many of the metal complexes, together with chloroiridium(III) complexes, act through the redox-arbitrated mode of action [14]. These compounds disturb the cell oxidation–reduction homeostasis by metal cations or ligands’ redox centers, or their activity is associated with binding interactions with redox biological macromolecules. With this assumption, redox-moderating reactions are frequently catalytic [15] and such redox-active compounds, including antineoplastic chloroiridium(III) complexes, can be classified as catalytic metallodrugs [16]. That is why chloroiridium(III)-containing complexes have emerged as promising capable candidates in anticancer drug discovery [17].

Iridium-based complexes, including chloroiridium(III) compounds, can inhibit cell growth through different biological interactions, act as efficient photosynthesizers and biocatalysts with selectivity, limited side effects and a greater kinetical magnitude of ligand substitution than platinum-based scaffolds, thus displaying a growing potential as anticancer agents over the course of time. This review provides readers with an outline of recent information on the development of different types of antitumor chloroiridium(III) complexes as a suitable strategy for the progress of novel biologically active metal complexes. To highlight the influence of the co-ligands, the chloroiridium(III)-based complexes have been discussed in descending order of the number of chloride ligands from 4 in tetrachloridoiridium(III) to 1 in monochloridoiridium(III) complexes with different biologically active ligands.

## 2. Tetrachloridoiridates(III)

**Figure 1:**
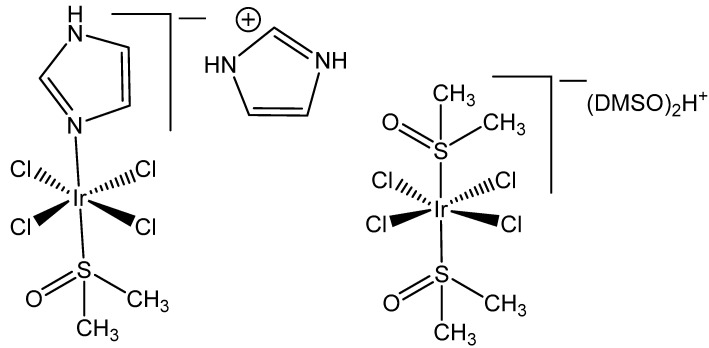
Structures of the tetrachloridoiridates(III) [ImH][trans-{IrCl_4_(DMSO)(Im)}] and [(DMSO)_2_H][trans-{IrCl_4_(DMSO)_2_}].

The ligand exchange reactions proceed generally more slowly for Ir(III) complexes than for their ruthenium(III) and rhodium(III) counterparts. The complexes, containing dimethyl sulfoxide (DMSO), NAMI A analog [ImH][trans-{IrCl_4_(DMSO)(Im)}], and the related iridium(III) complex [(DMSO)_2_H][trans-{IrCl_4_(DMSO)_2_}], shown at Figure 1, were found to be kinetically and biologically inert and stable [18]. Such findings have led to a general rule that the lack of chemical reactivity of many Ir(III) complexes will correlate with an absence of cytotoxicity on tumor cells, even at higher concentrations [19].

The conformational and structural variability of tetrachloridoiridium(III) complexes, containing DMSO, has been recently described by Marcon et al. [20]. The structures exhibited different rotamers of the *trans*-[IrCl_4_(DMSO)_2_]^−^ anion with the *trans*-DMSO ligands oriented in *anti-* and *gauche*-conformations. The effects of the solvent and intermolecular interactions on the conformational preferences of the anion have been discussed. The density functional theory (DFT) calculations have been used to investigate the conformations. It has been found that H-bonds between H_2_O and the DMSO complex stabilized the least stable *gauche* conformation form of the *trans*-DMSO complex. Thus, controlling the number of H-bond donors and acceptors and the amount of H_2_O is a good possibility to obtain information on the different solvatomorphs of these metallodrugs, associated with their biological activity.

**Figure 2:**
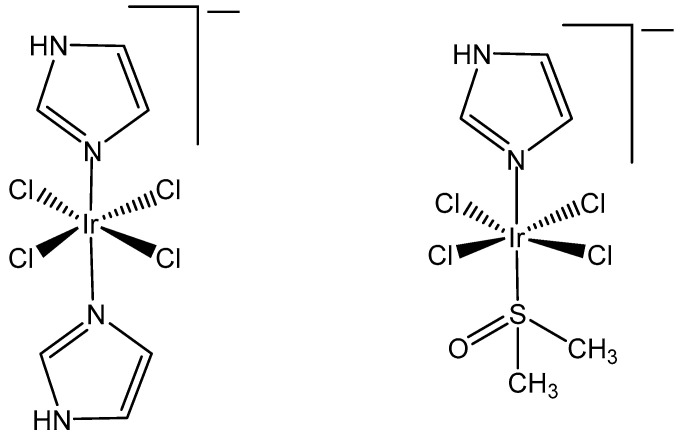
Structures of [Ir(Im)_2_Cl_4_]^−^ and [Ir(Im)(DMSO)Cl_4_]^−^.

It has been proven that the Ru(III) bis(imidazole) tetrachloride complex [Ru(Im)_2_Cl_4_]^−^ and its monodimethylsulfoxide counterpart [Ru(Im)(DMSO)Cl_4_]^−^ displayed very promising antineoplastic and antimetastatic effects connected with the hydrolysis of Cl^−^ ligands, while the tetrachloridoiridium(III) analogous complexes [Ir(Im)_2_Cl_4_]^−^ and [Ir(Im)(DMSO)Cl_4_]^−^ (Figure 2) did not exhibit pharmacological activity [19,20]. These complexes neither covalently modified DNA nor proteins nor inhibited the proliferation of the tested human cancer cell lines. Both [ImH][trans-{IrCl_4_(DMSO)(Im)}] and [(DMSO)_2_H][trans-{IrCl_4_(DMSO)_2_}] were stable in a physiological buffer (pH 7.4) at normal temperature during a period of days [19]. No indication for chloride ion exchange was found at temperatures of 70 °C over 24 h. The observed absence of significant inhibition of the tested cell lines, including human breast carcinoma MCF7, mouse breast carcinoma TS/A and fibrosarcoma HT-1080, by these tetrachloridoiridium(III) complexes, suggested that the initial Cl^−^/H_2_O exchange would most likely play a significant role in initiating the biopathway of the corresponding Ru(II) anticancer representatives, KP1019 and NAMI A. The rhodium(III) corresponding complexes [Na][trans-{RhCl_4_(Im)_2_] and [ImH][trans-{RhCl_4_(DMSO)-(Im)] were also basically inactive against selected tumor cells, but [ImH][trans-{RhCl_4_(DMSO)-(Im)] indeed caused the modest growth inhibition of primary MCa tumors implanted in mice.

It has been found that [Ir(Im)_2_Cl_4_]^−^ slightly modified the DNA structure in the solution and exhibited nearly negligible cytotoxic effects toward the referent A2780 tumor cell line. As the Ru analog showed a promising antitumor profile against the selected in vivo models, it is interesting to establish whether the respective tetrachloridoiridium(III) compound would retain at least partially these in vivo effects, which could offer good opportunities to clarify the mode of action of this kind of compounds [21].

**Figure 3:**
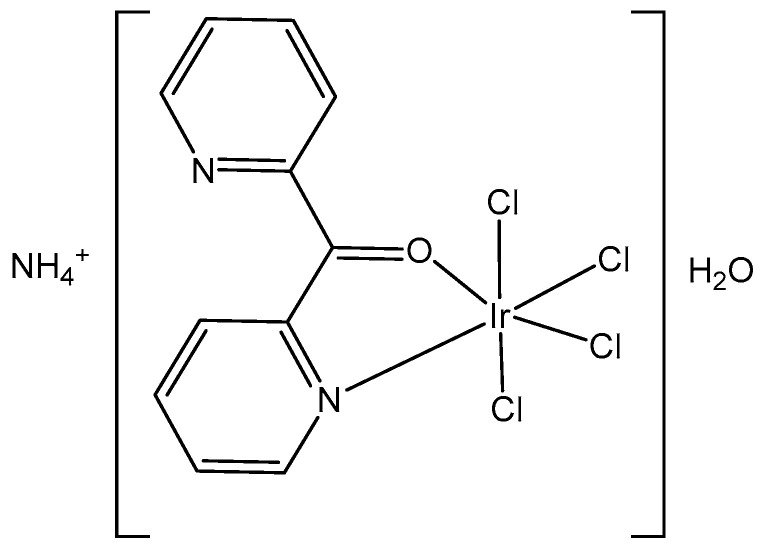
Structure of the complex NH_4_[IrCl_4_(py_2_CO)]·H_2_O.

In the study of [22], Masternak et al. recently obtained and characterized, by FTIR, ^1^H-, ^13^C-, ^15^N-NMR, UV–Vis, PL and elemental analysis, the octahedral complex NH_4_[IrCl_4_(py_2_CO)]·H_2_O (Figure 3), based on di-2-pyridylketone (py_2_CO). The compound was examined in vitro against LoVo (colorectal adenoma), MV-4-11 (myelomonocytic leukemia), MCF-7 (breast adenocarcinoma), and BALB/3T3 (normal fibroblasts) cells. The best results were obtained against MV-4-11 (IC_50_ = 35.8 ± 13.9 µg/mL) without toxic effects against normal BALB/3T3 cells, which pointed towards its selectivity as a potential antitumor agent. It has been suggested that this complex induced the probable formation of covalent bonds with DNA. Furthermore, the obtained tetrachloridoiridium(III) complex induced ROS, which, in synergy with hydrolysis promoting DNA bonding, could lead to cancer cell death. Furthermore, the biocatalytic activity of the complex against NADH (β-nicotinamide adenine dinucleotide disodium salt hydrate) has been determined. The complex exhibited a strong ability to interfere with the coenzyme NAD^+^/NADH, which is coupled to the induction of ROS in cells, consistent with the antineoplastic activity. According to the obtained data, the antiproliferative activity of the studied tetrachloridoiridium(III) complex has been derived from the synergistic effects of the hydrolysis and the oxidation–reduction reactions, generating ROS.

**Figure 4:**
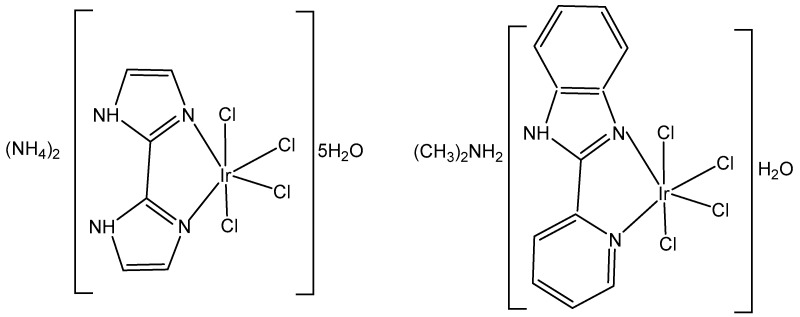
Structure of the complexes (NH_4_)_2_[IrCl_4_(κ^2^N,N′-H_2_biim)]_2_⋅5H_2_O and ((CH_3_)_2_NH_2_)[IrCl_4_(κ^2^N,N′-PyBIm)]⋅H_2_O.

The synthesis and the spectral (^1^H-, ^13^C- and ^15^N-NMR, FTIR, UV–Vis) characterization of the newly obtained anionic tetrachloridoiridium(III) complexes (NH_4_)_2_[IrCl_4_(κ^2^N,N′-H_2_biim)]_2_⋅5H_2_O and ((CH_3_)_2_NH_2_)[IrCl_4_(κ^2^N,N′-PyBIm)]⋅H_2_O, Figure 4, have been recently presented by the same group [23]. The structural analysis of iridium complexes showed a slightly distorted octahedral geometry of metal ions, which was built by one chelating κ^2^N,N′-2,2′-biimidazole (H_2_biim) or 2-(2′-pyridyl)benzimidazole (PyBIm) ligands and four chloride anions.

The cytotoxicity of the complexes (NH_4_)_2_[IrCl_4_(κ^2^N,N′-H_2_biim)]_2_⋅5H_2_O and ((CH_3_)_2_NH_2_)[IrCl_4_(κ^2^N,N′-PyBIm)]⋅H_2_O has been determined by an MTT assay against colorectal adenoma (LoVo), myelomonocytic leukemia (MV-4–11), and breast adenocarcinoma (MCF-7) cell lines, as well as against a healthy mouse fibroblast (BALB/3T3) cell line. The stronger binding affinity of (NH_4_)_2_[IrCl_4_(κ^2^N,N′-H_2_biim)]_2_⋅5H_2_O to DNA and HSA has been explained by the changes in the number of H-bonds that were potentially formed with the target biomolecules. In addition, a higher reactivity towards NADH and lower reactivity towards GSH have been observed for ((CH_3_)_2_NH_2_)[IrCl_4_(κ^2^N,N′-PyBIm)]⋅H_2_O. A moderate cytotoxic activity against LoVo, MV-4–11, MCF-7cell lines and a lack of toxicity towards normal BALB/3T3 cells have been detected. The cytotoxicity of ((CH_3_)_2_NH_2_)[IrCl_4_(κ^2^N,N′-PyBIm)]⋅H_2_O (IC_50_ 40.75 ± 13.00 against the most sensitive myelomonocytic leukemia (MV-4-11) cell line can be associated with a weaker interaction with DNA, HSA or GSH and a stronger reactivity towards NADH [23].

## 3. Trichloridoiridium(III) Complexes

**Figure 5:**
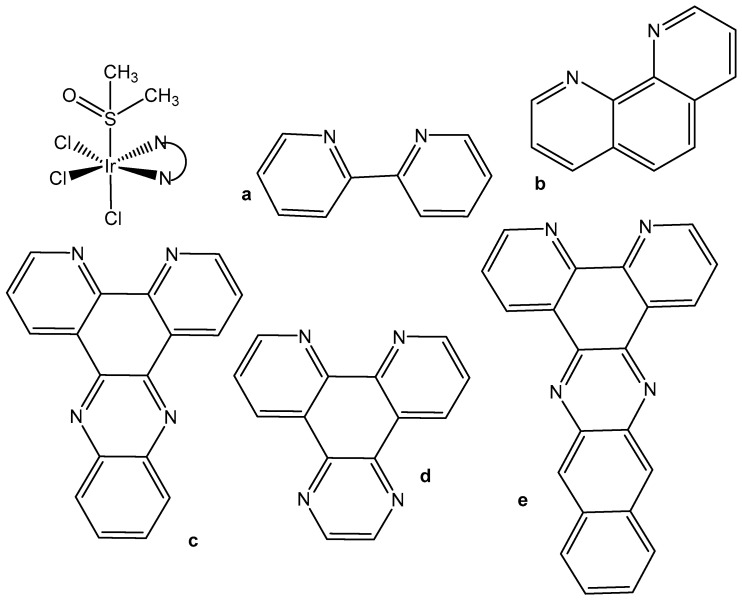
Structures of Ir(III) complexes fac-[IrCl_3_(DMSO)(pp)] containing the polypyridyl ligands bpy (2,2′-bipyridyl) (**a**), phen (1,10-phenanthroline) (**b**), dpq (dipyrido [3,2-d:2′,3′-f]quinoxaline) (**c**), dppz (dipyrido [3,2-a:2′,3′-c]phenazine) (**d**), and dppn (benzo[i]dipyrido [3,2-a:2′,3′-c]phenazine (**e**).

In spite of the promising but sporadic findings, systematic research on structure–activity relationships or probable cellular modes of action of trichloridometal(III) complexes had long been missing. One of the best approaches to improve the activity of metal complexes is to involve biologically active ligands, which are able to interact with the possible biotargets. Polypyridyl ligands (pp) are principally very appropriate in this respect owing to their potential DNA interactions and their impact on the oxidation–reduction and hydrophobic properties of the produced complexes. The neutral trichloridoiridium(III) complexes fac-[IrCl_3_(DMSO)(pp)], containing the polypyridyl ligands, such as bpy (2,2′-bipyridyl), phen (1,10-phenanthroline), dpq (dipyrido [3,2-d:2′,3′-f]quinoxaline), dppz (dipyrido [3,2-a:2′,3′-c]phenazine), and dppn (benzo[i]dipyrido [3,2-a:2′,3′-c]phenazine (see Figure 5), have been prepared and tested against the human tumor cell lines MCF-7 and HT-29 (colon carcinoma) and towards immortalized human embryonic kidney HEK-293 cells, by using the crystal violet assay [24,25]. The newly obtained complexes reacted slowly with compounds containing soft S donor atoms (e.g., N-acetylmethionine) but did not react with the guanine base of 5′-GMP^2−^. Their cytotoxicity and cellular impact have been studied in detail. Despite their low levels of cellular uptake, the polypyridyl complexes were potent in vitro cytotoxic agents toward the investigated human cell lines MCF-7 and HT-29. A structure–activity relationship between the compounds’ activity and the surface area of the polypyridyl ligands was clearly apparent when comparing the IC_50_ values, which were found to be dependent on the size of the polypyridyl ligands. 

The facial trichloridoiridium(III) complexes (Figure 5) were prepared by the sequential treatment of a methanol solution of IrCl_3_·3H_2_O with the respective pp ligand followed by DMSO and were indefinitely stable in aqueous and methanol solutions but rapidly isomerized to *fac/mer* mixtures in a CH_2_Cl_2_ solution under room light irradiation [24]. No DMSO/D_2_O or Cl/D_2_O substitution was evident in the ^1^HNMR spectra of aqueous solutions of the complexes with pp = bpy, phen, dpq over a 24 h period. Due to the greater trans-effect of the S donor atom of DMSO in comparison to halide ligands, meridional isomers such as *mer*-[IrX_3_(DMSO)(phen)] (X = Cl, Br) could be synthesized by simply adding DMSO prior to the pp ligand [25]. The antiproliferative activities of the complexes *fac*-[IrCl_3_(-L)(pp)] (L = DMSO, H_2_O) towards MCF-7, HT-29 and HEK-293 cells were directly comparable with the size for their pp ligands. The following structure–activity relationships could be established for the IC_50_ values: (a) bpy > phen, dpq > dppz > dppn for L = DMSO, (b) phen > 5-MePhen > 5,6-Me_2_phen for L = DMSO, (c) L = H_2_O > DMSO, and (d) *mer* > *fac* for L = DMSO and pp = phen. Coordination of the pp ligands bpy–dppn in *fac*-[IrCl_3_(κS-DMSO)(pp)] had effectively no influence on their potency when compared to the free ligand molecules. This suggested that the inert and neutral {IrCl_3_(DMSO)} fragment may merely adopt a decorating function with respect to the pp ligands and may have little influence on either their cellular uptake and distribution or their possible interactions with target biomolecules. Noticeable increases in the DNA thermal denaturation temperature T_m_ due to increased stabilization of the double helix provided a straightforward means of estimating the strength of possible pp intercalation.

**Figure 6:**
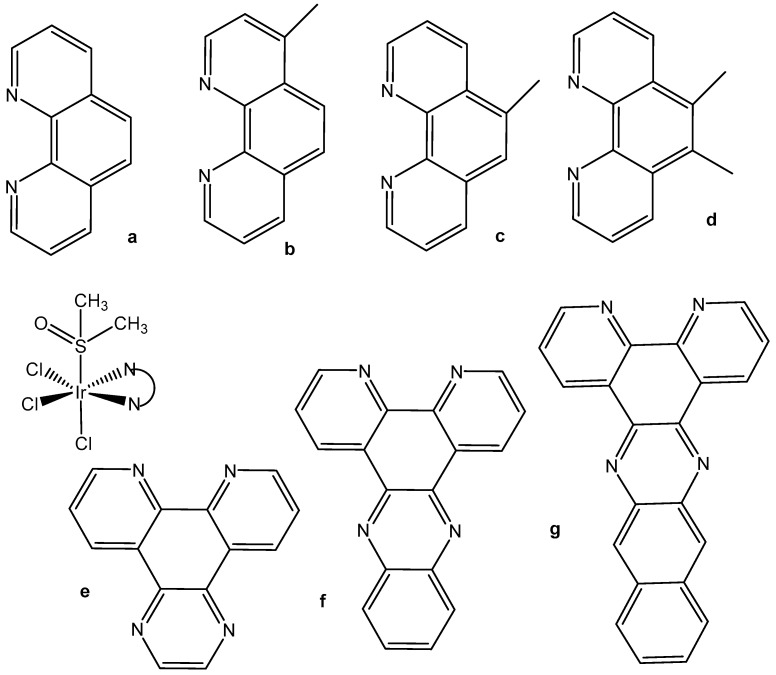
Heteroleptic diimine polypyridyl complexes and their N^ʌ^N ligands: phen (1,10-phenanthroline) (**a**), Me-phen (**b**,**c**), Me_2_-phen (**d**), dppz (dipyrido [3,2-a:2′,3′-c]phenazine) (**e**), dpq (dipyrido [3,2-d:2′,3′-f]quinoxaline) (**f**), and dppn (benzo[i]dipyrido [3,2-a:2′,3′-c]phenazine (**g**).

Despite the postulated inertness, other trichloridoiridium(III) complexes that exhibited relevant cytotoxic activity have been designed. The diimine complexes *fac*-[Ir(NˆN)(DMSO)Cl_3_] (Figure 6a–d) have shown antiproliferative properties toward the human cancer cell lines MCF-7 and HT-29 [26]. Since the order of activity of the trichlorido iridium complexes with methyl-substituted phenanthroline ligands (a < b, c < d), Figure 6, was in accordance with that of the free diimine ligands (phen < Me-phen < Me_2_-phen), the enhanced cytotoxic activity of these diimine complexes was believed to originate from the lipophilicity of the ligands, which played a role in cellular uptake efficiency. The related complexes *fac*-[Ir(NˆN)(DMSO)Cl_3_] (e–g) also displayed similar ligand-dependent antiproliferative activity (e < f < g), Figure 6, toward MCF-7 and HT-29 cells. Their cellular uptake efficiency was analyzed by graphite furnace atomic absorption spectroscopy (GF-AAS), and the results showed that the higher activity of the complex g (Figure 6) was related to its more efficient uptake. In addition to the diimine ligand, the solvent and halide ligands, as well as the stereo-structure, all affected the activity of the complexes of the type [Ir(NˆN)(solvent)X_3_]. The IC_50_ values of the aqua complexes were larger than those of their DMSO counterparts, whereas those of the chloride complexes were larger than those of the bromide analogs, which might be a consequence of the nature of the displaced ligands. Also, the meridional isomers of the trichloridoiridium(III) complexes [Ir(phen)(DMSO)X_3_] (X = Cl, Br) were significantly less cytotoxic than their facial counterparts. In contrast to the bpy–dppn series, coordination of the 4-, and 5-methyl, and 4,7- and 5,6-dimethyl-substituted phenanthroline ligands in the complexes *fac*-[IrCl_3_(DMSO)(phen*)] (Figure 6) led to a 1.5- to 7.5-fold decrease in potency with respect to the free ligands. The observed IC_50_ values for MCF-7 and H-29 cells [26] were, however, all significantly lower than those for fac-[IrCl_3_(DMSO)(phen)] [24].

In spite of the kinetic inertness of both the DMSO complexes and their aqua counterparts [25], replacement of the DMSO ligand by H_2_O molecules surprisingly led to a significant deterioration in the antiproliferative properties of phen and dpq complexes of the type *fac*-[IrCl_3_(L)(pp)]. The markedly lower activity of the aqua complexes suggested that the DMSO/H_2_O exchange could not play a significant role in the mechanism of action of the studied DMSO complexes. IC_50_ values of 5.5 and 4.3 μM indicated that *fac*-[IrBr_3_(H_2_O)(phen)] was significantly more active towards MCF-7 and HT-29 cells than its facial trichlorido counterparts, and markedly higher IC_50_ values of 20.3 and 16.8 μM were recorded for the isomer *mer*-[IrCl_3_(DMSO)(phen)] (Figure 6) in comparison to *fac*-[IrCl_3_(DMSO)(phen)]. The inert nature of the trichloridoiridium(III) complexes suggested that the observed differences in activity (*mer* < *fac*, H_2_O < DMSO, Cl < Br) would most likely be related to differences in their hydrophobicity and the resulting levels of cellular uptake. The antiproliferative activity of the complexes was strongly selective toward MCF-7 and HT-29 cancer cells over normal HFF-1 and immortalized HEK-293 cells. Therefore, the trichloridoiridium complexes could represent a novel class of potent anticancer agents with pronounced selectivity toward adherent cancer cells and lymphoma relative to normal cells. Their distinct and complex pharmacodynamic profile included high ROS levels, apoptosis induction, and the inhibition of cell respiration.

**Figure 7:**
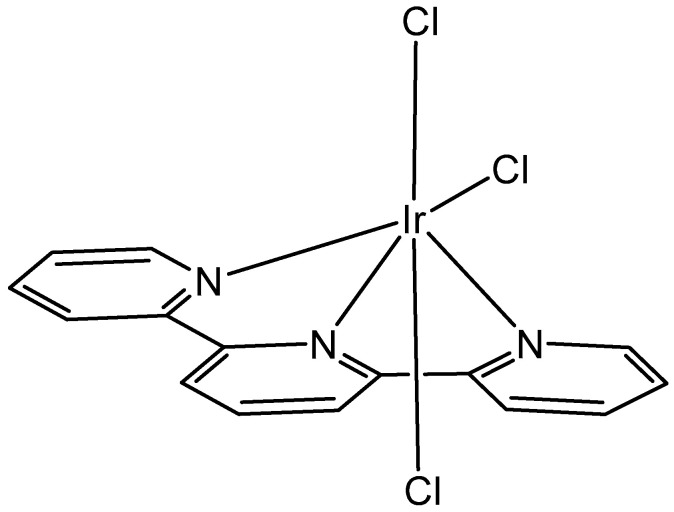
The structure of the complex [Ir(tpy)Cl_3_].

The photochemically active complex [Ir(tpy)Cl_3_], Figure 7, has primarily been employed as a precursor to create more complicated inert polypyridyl iridium(III) complexes. The compound [Ir(tpy)(ppy)Cl]^+^ (tpy = 2,2′:6′,2″-terpyridine and ppy = 2-phenylpyridine) has been used for the synthesis of luminophore architecture [Ir(N^N^N)(C^N)L]^+^ (N^N^N = tridentate polypyridyl ligand, C^N = 2-phenylpyridine derivative, and L = monodentate anionic ligand), which offered the stability of tridentate polypyridyl coordination along with the tunability of three independently variable ligands [27]. Additionally, a series of polypyridyl iridium(III) complexes that contain labile chlorido ligands, [{Ir(tpy)Cl}_2_{μ-bb*_n_*}]^2/4+^ {Cl-Mbb*_n_*; tpy = 2,2′:6′,2′′-terpyridine; and bb*_n_* = bis[4(4′-methyl-2,2′-bipyridyl)]-1,*n*-alkane (*n* = 7, 12 or 16)} have been synthesized and examined by Pandrala et al. [28].

The cellular impact of the above mentioned complexes *fac*-[IrBr_3_(H_2_O)(phen)] and *mer*-[IrCl_3_(tpy)] has been studied for non-adhesive Burkitt-like lymphoma cells (BJAB) [25]. The *fac*-[IrBr_3_(H_2_O)(phen)] and *mer*-[IrCl_3_(tpy)] complexes were active cytotoxic agents as established by low IC_50_ values of 0.32/0.33 and 0.13/0.26 μM against MCF-7 and HT-29 adenocarcinoma cells [25]. Dose-dependent proliferation inhibition with IC_50_ = 5.0 and 0.5 μM, respectively, has been found for the BJAB cells after 24 h treatment with tribromido *fac*-[IrBr_3_(H_2_O)(phen)] and triclorido *mer*-[IrCl_3_(tpy)] complexes [25]. DNA fragmentation was considered an indicator of apoptosis and was observed at high BJAB cells levels after 72 h exposures to the iridium complexes [25]. Much higher cytotoxicity for *mer*-[IrCl_3_(tpy)] has been detected compared to *fac*-[IrBr_3_(H_2_O)(phen)]. Additionally, Annexin V/propidium iodide assays demonstrated that the 5,6-dimethylphenanthroline complex *fac*-[IrCl_3_(DMSO)(5,6-Me_2_phen)] caused a significant inhibition of Jurkat leukemia cell proliferation and invoked extensive apoptosis but negligible necrosis. The percentages of Jurkat cells exhibiting high levels of reactive oxygen species correlated with the percentages of cells undergoing apoptosis [26]. The higher activities of the trichlorido-substituted rhodium(III) complexes were accompanied by pronounced selectivity towards human cancer cells in comparison with immortalized HEK-293 cells [29].

**Figure 8:**
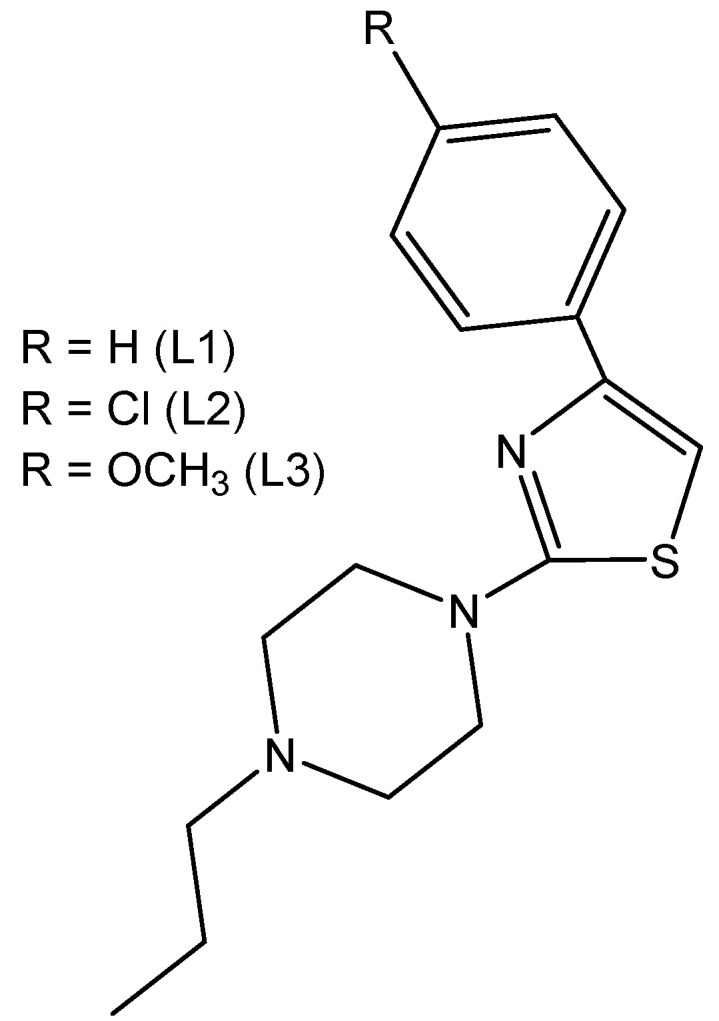
Structures of the derivatives of 1-[2-thiazol-4-yl-(4-substitutedphenyl)]-4-n-propylpiperazines.

The thiazole-based complexes Ir(L1)(H_2_O)_2_Cl_3_, Ir(L2)(H_2_O)_2_Cl_3_ and Ir(L3)(H_2_O)_2_Cl_3_ of the organic derivatives of 1-[2-thiazol-4-yl-(4-substitutedphenyl)]-4-n-propylpiperazines, shown in Figure 8, have been synthesized and characterized [30]. It has been proven that, in all the studied complexes, the coordination appeared through the N atom from the thiazole ring. The interactions of the complexes with CT-DNA have been studied. In vitro DNA interactions have confirmed that Ir(L3)(H_2_O)_2_Cl_3_ had the strongest binding affinity among the newly synthesized trichloridoiridium(III) complexes. The authors have investigated the effect of complexation on the antineoplastic activity of the new compounds against the human LN-229 glioma and MDAMB-231 breast cancer cells. Cytotoxicity studies have shown that, the complexation process in Ir(L1)(H_2_O)_2_Cl_3_ increased the activity towards the glioblastoma LN-229 cells. In terms of breast cancer cell lines, it was found that coordination with Ir(III) significantly improved the activity of L2 and L3 ligands. These results have supported the promising therapeutic potential of trichloridoiridium(III) complexes with thiazole-based ligands.

## 4. Dichloridoiridium(III) Complexes

**Figure 9:**
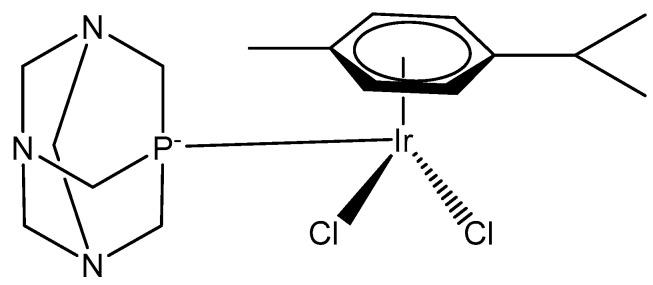
Iridium(III) RAPTA-C analog.

The dichloridoiridium(III) half-sandwich complex [(η^5^-C_5_Me_5_)IrCl_2_(pta)] of phosphine 1,3,5-triaza-7-phosphatricyclo[3.3.1.1] decane (pta), Figure 9, has been screened against a range of human tumor cell lines [31,32]. The complex is an analog of the antimetastatic agent [(η^6^-cymene)RuCl_2_(pta)] (RAPTA-C).

Along with the cytotoxicity, the ability to inhibit cathepsin B in vitro in comparison to the antimetastatic compound NAMI-A, has also been screened. Cathesin B is a lysosomal papain-family cysteine protease, which has been implicated in tumor progression and metastasis processes that can be prevented by RAPTA compounds. In contrast to RAPTA-C (IC_50_ = 2.5 μM), its Ir(III) analog proved to be inactive (IC_50_ > 300 μM), despite its relatively rapid aquation and soft character. In order to build up a rational for the observed differences, DFT calculations of the complex adducts with N-acetyl-L-cysteine-*N*′-methylamide, a mimic for the Cys residue in the cathepsin B active site, have been performed to provide insights into binding thermodynamics in solution. Initial structure–activity relationships have been defined with the calculated binding energies of the M–S bonds, correlating well with the observed inhibition properties. IC_50_ values > 500 μM for the Ir(III) complex towards A2780 cells were determined. Inactivity (IC_50_ > 400 μM) was also established for the monochlorido complexes with the general formula [(η^5^-C_5_Me_5_)MCl(pta)_2_]^+^ (M = Rh, Ir). A lower value of 349 μM, comparable with that of 353 μM for RAPTA-C itself, was recorded for [(η^5^-C_5_Me_5_)IrCl_2_(mpta)] (mpta = 1-methyl1,3,5-triaza-7-phosphatricyclo-[3.3.1.1] decane) against A2780 cells [32].

### 4.1. Dichloridoiridium(III) Complexes with Cyclopentadienyl Ligands

Organometallic iridium(III) complexes with a d^6^ electron configuration are hypothetically inert. Definitely, [Ir(H_2_O)_6_]^3+^ exchanges ligands very slowly. Nevertheless, the introduction of a cyclopentadienyl ligand Cp^x^ strongly increases the ligand exchange rate. The half-sandwich (cyclopentadienyl)iridium(III) complexes Ir–Cp^x^ and their derivatives represent one of the basic structural types of Ir(III) complexes with pseudo-octahedral geometry consisting of η^5^-coordinated substituted cyclopentadienyl anions Cp^x^ occupying three of the six positions. The other three coordination sites can be occupied either by three monodentate ligands or by a bidentate chelating ligand combined with a monodentate one. Complexes containing three monodentate ligands usually belong to the family of dichloridoiridium complexes of the general formula [Ir(η^5^-Cp^x^)Cl_2_(L)]^0/+^, with two chlorido ligands and a third monodentate ligand (L) coordinated through various donor atoms (see below). The second type is dominantly represented by monochloridoiridium complexes of the general formula [Ir(η^5^-Cp^x^)Cl(L^L)]^0/+^, where L^L represents a bidentate-coordinated chelating ligand (discussed in the next section). Although the structure of Ir–Cp^x^ complexes is rather strict (with a Cp^x^ ligand and three additional positions), various synthetic approaches can be used to modify their biochemical and biological properties, including reactivity and antineoplastic activity.

Principally, contrasting Ru(II) and Os(II), it is difficult to stabilize Ir(III) with arene ligands, which is why, in most cases, cyclopentadienyl and especially pentamethylcycopentadienyl ligands (Cp*) have been used. Studies have shown that these complexes have exhibited strong electron donor characteristics, enhancing the rate of ligand exchange and hydrolysis, thus easily forming aqua complexes [33]. They have shown capacity to generate low ROS levels, to influence mitochondrial membrane potential and to induce apoptosis. Many (cyclopentadienyl)iridium(III) compounds with a number of different structures, representing a well-established family of organometallic drug candidates, were found to display potent anticancer effects against many tumor cell lines in low micromolar concentrations and were believed to be invincible in cells [34,35,36]. Along with this, most Ir(III) complexes with the general formula [(Cp*(Ir(L^L)Z]PF_6_, where Cp* is a cyclopentadienyl group itself or its derivatives, Z is a leaving group and L^L is a chelating ligand, have shown excellent physiochemical properties [37,38].

**Figure 10:**
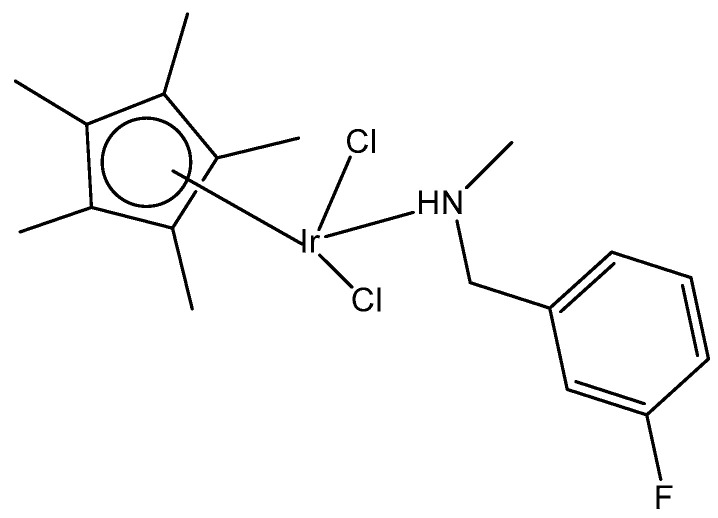
Dichloridoiridium(III) complex with 3-fluoro-*N*-methylbenzylamine ligand.

A half-sandwich dichloridoiridium(III) complex, containing secondary amine 3-fluoro-N-methylbenzylamine as the N-monodentate ligand, has been obtained by the reaction of one equivalent of [(η^5^-Cp*)IrCl_2_]_2_ (Cp* = pentamethylcyclopentadienyl) with two equivalents of 3-fluoro-N-methylbenzylamine in very good yield [39]. The structure of the dichloridoiridium complex (Figure 10) has been confirmed by X-ray crystallography, ^1^H-NMR, ^13^C-NMR spectroscopy, and elemental analysis. The obtained complex adopted a piano-stool configuration, with Cp* acting as the seat and 3-fluoro-*N*-methylbenzylamine ligand and chloride groups as the legs. The cyclometallation reaction of iridium(III) complexes could occur when tertiary amines were employed [40]. As a result, it seemed that the production of monoligated complexes in this system was ascribed to the small size of the secondary amines compared to tertiary amines.

**Figure 11:**
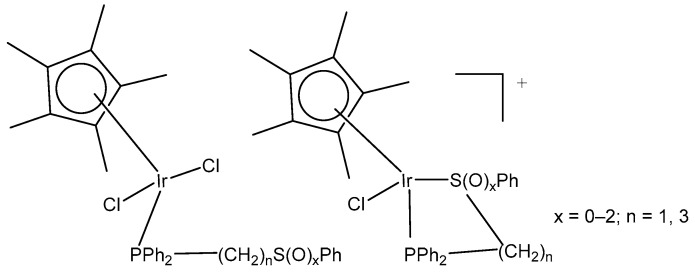
Iridium(III) complexes with κP- and κP,κS-Ph_2_P(CH_2_)_n_S(O)_x_Ph ligands.

Complexes (η^5^-C_5_Me_5_)Ir(III), containing Ph_2_P(CH_2_)_n_S(O)_x_Ph (n = 1–3; x = 0–2) with κP- and κP,κS-coordination (Figure 11) and their high antitumor potential in a low µM range, have been described by Ludwig et al. [41]. The complexes with κP- and κP,κS-Ph_2_P(CH_2_)_n_S(O)_x_Ph ligands have been found to be more active compared to cisplatin against tumor cell lines from different origins, such as A253, 518A2, SW480, 8505C and MCF-7, showing apoptotic induction.

Recently, the authors have reported the synthesis and the theoretical and experimental characterization of the (pentamethylcyclopentadienyl)chloridoiridium(III) complex with a Ph_2_PCH_2_CH_2_SPh-κP,κS ligand [42]. The in vitro antineoplastic activity of the complex (η^5^-C_5_Me_5_)Ir(III)Cl{Ph_2_PCH_2_CH_2_SPh-κP,κS}][PF_6_] against human breast adenocarcinoma MCF-7, colon adenocarcinoma SW480, melanoma 518A2, thyroid carcinoma 8505C and submandibular carcinoma A253 cells and their mechanism of action against 8505C have been investigated. Caspase-induced apoptosis, cell cycle arrest in 8505C cells and a strong reduction in ROS/RNS have correlated with the observed cytotoxic effects.

**Figure 12:**
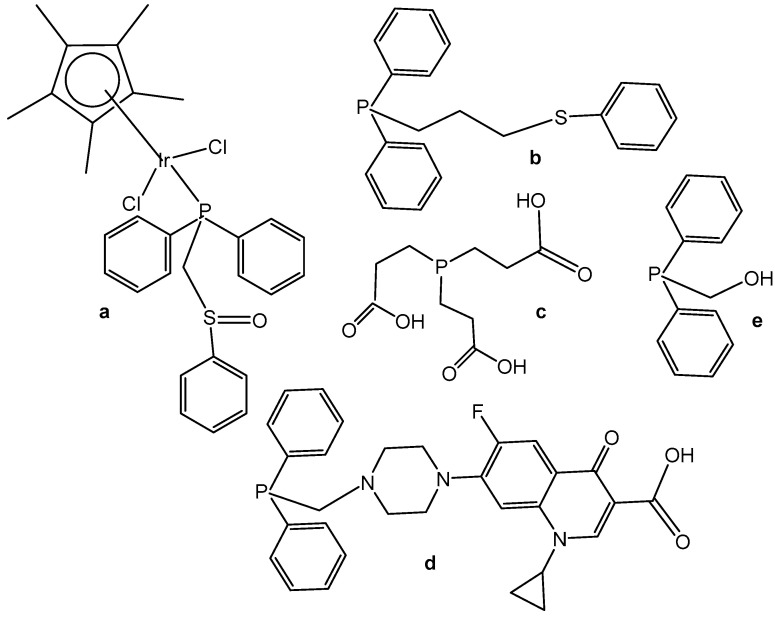
Structural formulas of electroneutral complex [Ir(η^5^-Cp*)Cl_2_(phs^1^)] (**a**) and analogs P-donor ligands phs^2^, tcep, psf and poh (**b**–**e**), respectively.

Neutral and cationic dichloridoiridium(III) complexes with diphenylphosphano-functionalized methyl-phenyl sulfides, sulfoxides, and sulfones Ph_2_PCH_2_S(O)*_x_*Ph (*x* = 0–2) were, in most cases, less potent than cisplatin for the panel of cell lines. New dichloridoiridium(III) complexes with Ph_2_PCH_2_S(O)*_x_*Ph ligands have been synthesized and characterized analytically and spectroscopically [43]. The biological potential of the neutral and cationic dichloridoiridium(III) complexes has been established in vitro against A253, MCF-7, 518A2 and cisplatin-resistant (thyroid carcinoma 8505C, colon carcinoma SW480) cell lines. Only the complex [Ir(η^5^-Cp*)Cl_2_(phs^1^)] (Figure 12a), where phs^1^ is diphenyl[(phenylsulfinyl) methyl]phosphane, has exceeded cisplatin against 8505C (IC_50_ = 3.5 μM) and SW480 (IC_50_ = 2.3 μM). Additional experiments have confirmed that apoptosis and autophagic cell death contributed to the drug’s antitumor action. While apoptosis and autophagy were detected in 8505C cells, the lower level of ROS and RNS was found in 8505C cells treated with [Ir(η^5^-Cp*)Cl_2_(phs^1^)] compared with nontreated ones. Cell cycle analysis has shown that [Ir(η^5^-Cp*)Cl_2_(phs^1^)] induced an increase in apoptosis-related sub-G_1_ cells with fragmented DNA at the expense of the G_1_, S and G_2_/M cell cycle phases, suggesting that apoptosis was the dominant type of cell death for the studied dichloridoiridium complex.

A correlation between the oxidation state of the S atom (*x* = 0–2) and the cytotoxicity of the complexes (Figure 12) has been found [43,44]. The same research team developed a series of analogous complexes of the type [Ir(η^5^-Cp*)Cl_2_{Ph_2_PCH_2_CH_2_CH_2_S(O)*_x_*Ph-κP}] (*x* = 0–2) and [Ir(η^5^-Cp*)-Cl{Ph_2_PCH_2_CH_2_CH_2_S(O)*_x_*Ph-κP,κS}][PF_6_] (*x* = 0, 1), involving similar ligands with a longer alkyl chain (propyl) [44]. Among them, the complex [Ir(η^5^-Cp*)Cl_2_(phs^2^)] (Figure 12b), where phs^2^ = diphenyl[3-(phenylsulfanyl)propyl]phosphane, exceeded the cytotoxic efficacy of cisplatin (IC_50_ = 0.2–0.6 μM). In vitro studies against human thyroid carcinoma 8505C, submandibular carcinoma A253, breast adenocarcinoma MCF-7, colon adenocarcinoma SW480, and melanoma 518A2 cell lines have provided evidence for the high biological potential of the tested neutral and cationic Ir(III) complexes. Apoptosis was identified in 8505C cells as the cell death mechanism. In another study the dichloridoiridium complex [Ir(η^5^-Cp*)Cl_2_(tcep)] (Figure 12c), where tcep is tris(2-carboxyethyl) phosphane, has been found to be more active (IC_50_ = 7.8 μM) towards triple negative breast cancer MDA-MB-231 cells than its Rh(III) analog (IC_50_ = 67 μM) and cisplatin (IC_50_ = 61 μM) [45].

Half-sandwich Ir(III) compounds containing phosphines derived from fluoroquinolones can be outstanding examples for the design of new therapeutics. A series of half-sandwich dichloridoiridium(III) complexes were obtained from various phosphane ligands by Kozieł et al. in several studies [46,47,48]. The dichloridoiridium(III) complex [Ir(η^5^-Cp*)Cl_2_(pcfx)] (Figure 12d), where pcfx is phosphane bearing fluoroquinolone ciprofloxacin, has exceeded (IC_50_ = 11.8 μM) the antiproliferative activity of cisplatin (IC_50_ > 100 μM) against human prostate carcinoma DU-145, a cell line that was also highly sensitive to various congenators of [Ir(η^5^-Cp*)Cl_2_(pcfx)]. The complex [Ir(η^5^-Cp*)Cl_2_(pcfx)] (Figure 12d) was highly selective towards the tested tumor cells over normal HEK-293 cells. Additionally, the investigated phosphane-derived dichloridoiridium(III) complexes were effective against various multicellular (3D) tumor spheroids. Other complexes, which contained diphenylphosphanyl) methanol (poh) or its 4,4-bismethoxy derivative (Figure 12e), have shown only moderate or even no cytotoxic activity (IC_50_ = 22.5 μM in A549 lung cancer cells, calculated for Figure 12e). The complex [Ir(η^5^-Cp*)Cl_2_(pcfx)] (Figure 12d) was stable in DMSO-d_6_/D_2_O and DMSO/DMEM mixtures of solvents. Its analogous dichloridoiridium(III) complex ([Ir(η^5^-Cp*)Cl_2_(poh)] (Figure 12e), bearing monodentate P-donor ligands, was hydrolyzed in the presence of water, but the diaqua species formed reacted with various concentrations of chlorides, suggesting that dichloridoiridium(III) complexes were most likely intact when accumulated in cells. In order to investigate the ability of dichloridoiridium(III) complexes to interact with DNA (intercalation), to monitor the structural changes in DNA, and to evaluate the ability of studied complexes to HSA and apoTf binding, Kozieł et al. [46,48] have applied different approaches based on fluorescence spectroscopy, circular dichroism (CD), gel electrophoresis and molecular docking. The experimental results have indicated a mode of action different from DNA targeting, which is typical for Pt(II) drugs. The observed activity was connected with the higher level of ROS generation.

**Figure 13:**
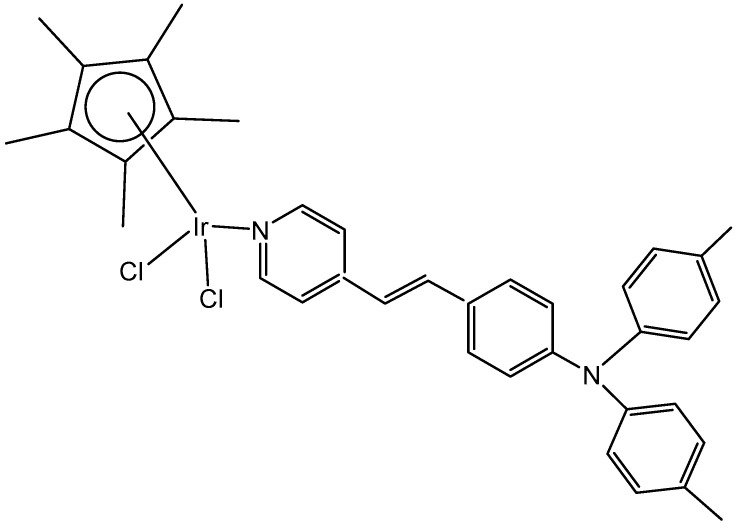
Structural formula of the dichloridoiridium(III) complex with monodentate N-donor ligand.

A series of half-sandwich Ir(III) complexes with triphenylamine-modified pyridines, used as a monodentate N-donor ligands, have been projected to act as luminescent agents for the intracellular tracking studies [49]. Ten triphenylamine-modified fluorescent half-sandwich dichloridoiridium(III) pyridine complexes with the general formula {[(η^5^-Cp^x^)Ir(L)Cl_2_]} have been prepared and found to display potential antiproliferative action, effectively inhibiting the A549 cell migration. The most active complex (Figure 13) showed moderate cytotoxic activity (IC_50_ = 26.7 μM in A549 cells) but exhibited excellent fluorescence performance in solution (15.2% quantum yield), allowing innovative in vitro and in vivo biological studies. Additionally, this dichloridoiridium(III) compound was able to arrest the cell cycle at the G_2_/M phase, catalyze NADH oxidation, reduce the mitochondrial membrane potential, induce an increase in the intracellular ROS levels, and exhibit a mechanism of oxidation. An in vivo anticancer assay confirmed that the dichloridoiridium(III) complex (Figure 13) was safer than the classical drug cisplatin.

Numerous half-sandwich dichloridoiridium(III) complexes of this type bearing monodentate N-donors (2-substituted-1,8-napthyridine ligands) [50], aroylthiourea ligands [51] or C-donor ligands, such as N-heterocyclic carbene-carbaboranyl complexes [52] showed no significant cytotoxicity against colorectal, ovarian, cisplatin-resistant ovarian and breast cancer cell lines. Similarly, an advanced metal–peptide conjugate coordinating numerous {Ir(η^5^-Cp*)Cl_2_} moieties through the benzimidazolium groups of a peptide was not effective against the tested human cancer cell lines [53]. The same lack of activity was found for their Ru(III), Rh(III) and Os(III) analogous complexes.

**Figure 14:**
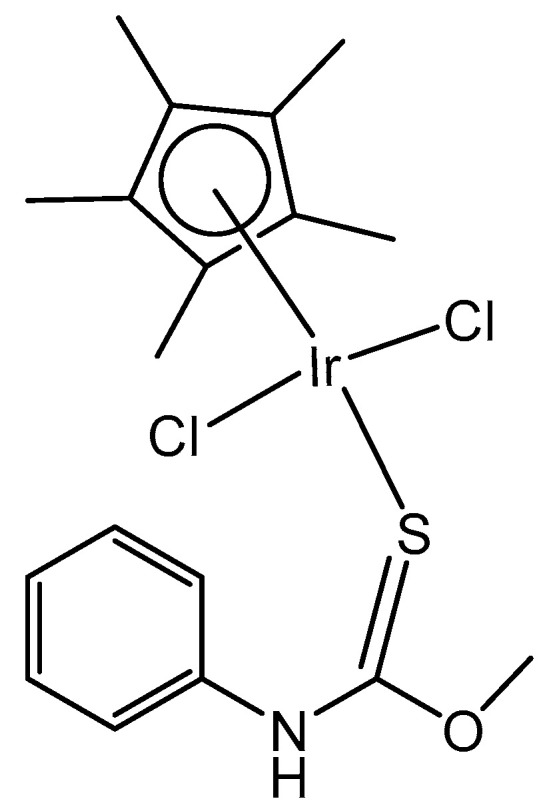
The complex [Ir(η^5^-Cp*)Cl_2_(pct^1^)] of the monodentate S-donor O-methyl phenylcarbamothioate (pct^1^) ligand.

The dichloridoiridium complex [Ir(η^5^-Cp*)Cl_2_(pct^1^)] (Figure 14) with a direct coordination of the thione sulfur to the iridium center, which involves monodentate S-donor O-methyl phenylcarbamothioate (pct^1^), has been studied for its antioxidant activity by the DPPH free radical scavenging method, where DPPH = 1,1-diphenyl-2-picryl-hydrazyl, [54] and has shown superior activity. Its DPPH radical scavenging activity (DRSA%) was 90% (tested at 1 mg mL^−1^ concentration), which was comparable with the reference compound using ascorbic acid (AA). In this regard, from the reported results, this kind of thiocarbamate complex could be of great interest to serve as antioxidant agents with great potential in the future. Similar antioxidant potency (92%) was reached by the electroneutral dichloridoiridium azido [Ir(η^5^-Cp*)Cl_2_(pct^1^)(N_3_)] complex with a bidentate N,S-coordinated pct^1^ ligand. The nature of the arene, the bonded ligands and the two facile chlorides in these complexes have strongly influenced their chemical, structural and biological activity. As the two chloride ions are facile, on the introduction of N_3_^ˉ^ as a co-ligand, new coordination could occur, which can lead to the substitution of the chloride ions entirely.

### 4.2. Dichloridoiridium(III) Cyclopentadienyl Dinuclear Complexes

**Figure 15:**
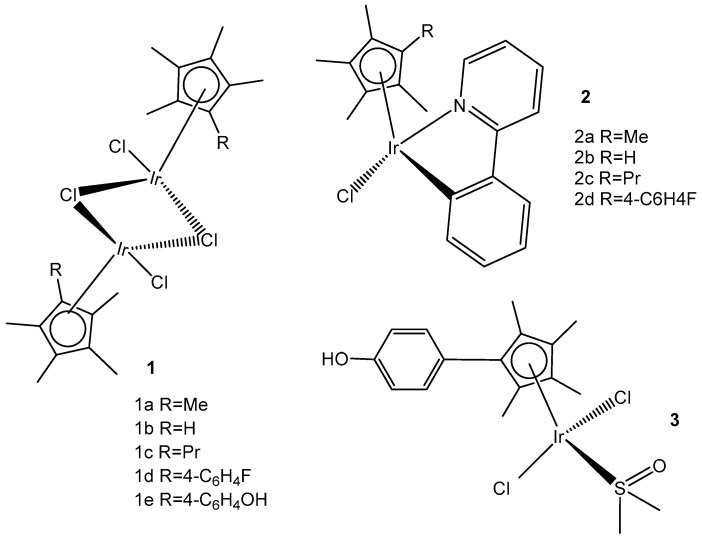
Structures of di- and mono-iridium complexes bearing variable tetramethylcyclopentadienyl ligands.

Two series of homologous di- and mononuclear iridium complexes, presented in Figure 15, [Ir(η^5^-C_5_Me_4_R)Cl(μ-Cl)]_2_ (R = Me, 1a; R = H, 1b; R = Pr, 1c; R = 4-C_6_H_4_F, 1d; R = 4-C_6_H_4_OH, 1e), their 2-phenylpyridyl mononuclear derivatives [Ir(η^5^-C_5_Me_4_R)(k*N*,k*C*PhPy)Cl] (2a–d), and the dimethylsulfoxide complex [Ir{η^5^-C_5_Me_4_(4-C_6_H_4_OH)}Cl_2_(κ*S*-Me_2_S=O)] (3) have been synthesized, characterized, and evaluated for their antiproliferative activity against a panel of human and rodent tumor cell lines, including mouse melanoma B16, rat glioma C6, breast adenocarcinoma MCF-7, colorectal carcinoma SW620 and HCT116, ovarian carcinoma A2780, and one primary, human fetal lung fibroblast cell line MRC5 [55]. Complexes 2b and 2d (Figure 15) have exhibited the best performance within the series. They have not affected the viability of primary mouse peritoneal cells, and their activity has arisen from the combined impact on cancer cells, consisting in proliferation inhibition, apoptotic cell death, and senescence induction, triggered by mitochondrial failure and the production of ROS and RNS. An evaluation of the effects of the modified Cp* ring by the replacement of one methyl with a range of substituents and their impact on the cytotoxicity has also been addressed. Particularly, the replacement of one methyl with a hydrogen atom has been proven sufficient to determine the fast degradation of the related mononuclear complex in aqueous media, which led to improved cytotoxicity.

Within the group of dichloridoiridium(III) complexes apart from multinuclear homometallic Ir(III) complexes, the heteronuclear iridium complexes are of great interest. The variation in the central metal has some profound effects on the observed properties of these complexes. The coordination of two different metal ions would significantly broaden the scope of their action mode with cells through different cytotoxic mechanisms. 

**Figure 16:**
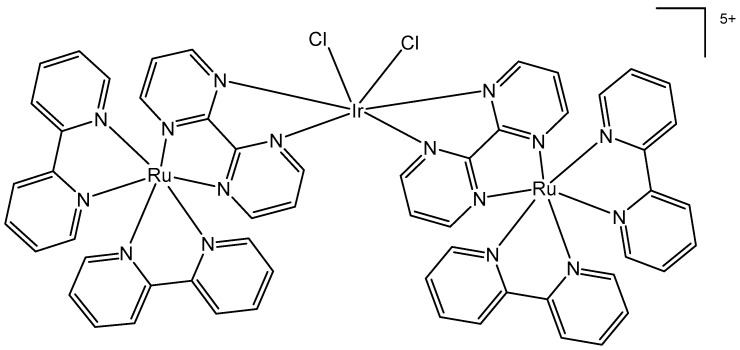
Structure of the trimetallic complex {[(bpy)_2_Ru(bpm)]_2_IrCl_2_}^5+^.

The bipyrimidine-bridged trimetallic complex {[(bpy)_2_Ru(bpm)]_2_IrCl_2_}^5+^, where bpy = 2,2′-bipyridine, and bpm = 2,2′-bipyrimidine (Figure 16) has been synthesized and characterized [56,57]. This kind of polymetallic complex combined the catalytical properties of iridium(III) with the photoactivity of ruthenium(II). A detailed characterization of spectroscopic, electrochemical, and spectroelectrochemical properties of the studied iridium(III)-based polymetallic complex has been described.

**Figure 17:**
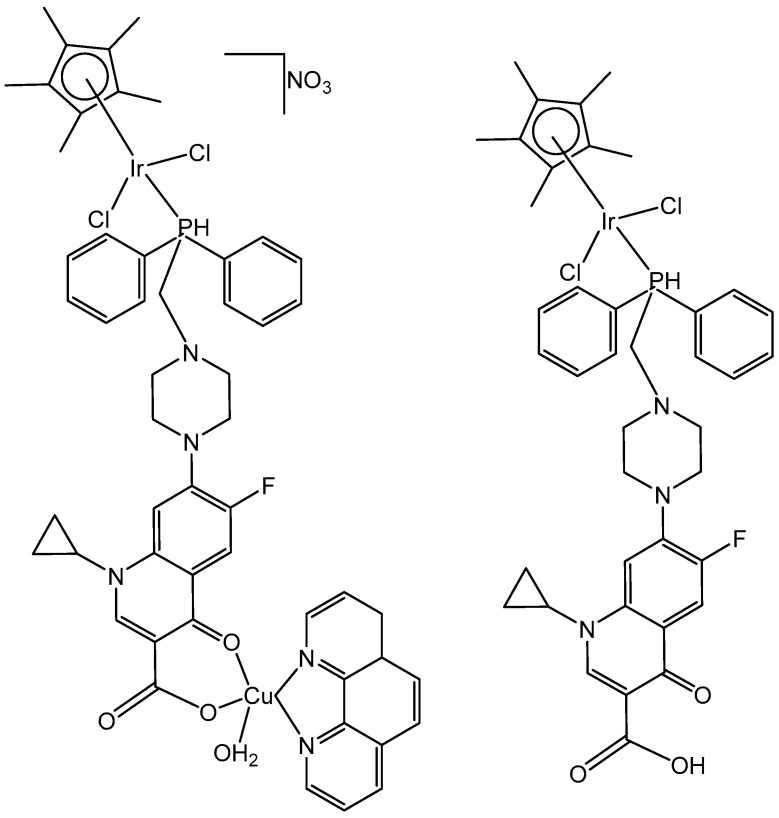
Iridium-copper heteronuclear complex [Ir(η^5^-Cp*)Cl_2_(μ-pcfx)Cu(phen)(H_2_O)]NO_3_ and its mononuclear analog.

Iridium-copper heteronuclear complexes have been obtained by the combination of phosphanes (P-coordinated to Ir) and fluoroquinolones (O,O-coordinated to Cu) as ligands [58]. Novel heteronuclear Ir(III)–Cu(II) coordination compounds ([Ir(η^5^-Cp*)Cl_2_Pcfx-Cu(phen)](NO_3_)·1.75(CH_3_OH)·0.75(H_2_O), [Ir(η^5^-Cp*)Cl_2_Pnfx-Cu(phen)](NO_3_)·1.75(CH_3_OH)·0.75(H_2_O), [Ir(η^5^-Cp*)Cl_2_Plfx-Cu(phen)](NO_3_)·1.3(H_2_O)·1.95 (CH_3_OH), [Ir(η^5^-Cp*)Cl_2_Psfx-Cu(phen)]), bearing phosphines derived from fluoroquinolones, namely, sparfloxacin (Hsfx), ciprofloxacin (Hcfx), lomefloxacin (Hlfx), and norfloxacin (Hnfx), have been synthesized and studied as possible anticancer chemotherapeutics. These complexes with piano-stool geometry, such as the best performing complex, [Ir(η^5^-Cp*)Cl_2_(μ-pcfx)Cu(phen)(H_2_O)]NO_3_ (Figure 17), were significantly more effective than cisplatin in various human cancer cell lines, whereas they were almost ineffective in non-cancerous HEK-293T cells. The cytotoxic effects of the compounds were assessed in vitro toward lung, breast, melanoma, and prostate tumor cell lines and one nontumor human embryonic kidney cell line. The heteronuclear Ir(III)/Cu(II) complexes displayed higher cytotoxicity than cisplatin against all cell lines (A549, MCF7, DU145) except for the WM2664 cell line. Especially the anticancer potency of the compound [Ir(η^5^-Cp*)Cl_2_(μ-pcfx)Cu(phen)(H_2_O)]NO_3_ in DU145 cells is noteworthy not only because of the extremely low IC_50_ value in the low-picomolar range (IC_50_ = 1.3 × 10^−6^ μM for 24 h exposure time), but also because of the unusual recovery of treated cells (IC_50_ = 125.7 μM for 24 h exposure + 48 h recovery time in a drug-free environment). Similar results were obtained for this compound and its analogs in MCF-7 cells, while the opposite effect was observed in A549 cells (IC_50_ = 35.5 nM for a 24 h exposure time, and IC_50_ = 0.4 nM for a 24 h exposure + 48 h recovery time). The complex [Ir(η^5^-Cp*)Cl_2_(μ-pcfx)Cu(phen)(H_2_O)]NO_3_ has also been loaded into liposomes. Importantly for future studies in the field of heterometallic anticancer Ir–Cp^x^ complexes, this complex exhibited markedly higher antiproliferative activity in cancer cells than its mononuclear analog, shown in Figure 17 [59]. It can be effectively accumulated inside human lung adenocarcinoma and human prostate carcinoma cells with selective localization in nuclei. The cytometric analysis has shown the dominance of apoptosis over the other cell death types.

## 5. Monochloridoiridium(III) Cyclopentadienyl Complexes

Monochlorido organoiridium(III) complexes, mainly cyclopentadienyl complexes, have shown great potential as anticancer agents. This type of organoiridium(III) complex is represented foremost by monochlorido complexes of the general formula [Ir(η^5^-Cp^x^)Cl(L^L)]^0/+^, where L^L denotes a bidentate-coordinated chelating ligand. The development of monochloridoiridium(III)–Cp^x^ complexes involving extended cyclopentadienyl ligands (for instance Cp^xph^, Cp^xbiph^ etc.) and C,N-; N,N-, etc., donor chelating ligands, or kinetically stable monodentate ligands (for example, pyridine or its derivatives) appears to be an appropriate strategy for the design of novel biologically active iridium complexes. Recent studies have reported half-sandwich monochloridoiridium complexes bearing ligands with other chelating modes, such as the P^N, P^P, P^O, C^C, C^O, etc., which exhibited various redox mechanisms of action. Furthermore, certain smaller counter anions, for instance, Cl^−^, PF_6_^−^, BF_4_^−^, CF_3_SO_3_^−^, with better coordination ability, have been found to contribute to the chemical reactivity and antineoplastic activity of the complexes. As shown above and as further discussed, the mechanism of action of anticancer monochloridoiridium(III)–Cp^x^ complexes, although still not completely understood, in many cases, differs from that of Pt(II) drugs and from those of the structurally analogous Ru(II), Ru(III), Rh(III), Os(II) and Os(III) half-sandwich complexes, which highlights their potential for novel therapeutic approaches in anticancer drug discovery.

### 5.1. Monochloridoiridium(III) Cyclopentadienyl Complexes with C,N-Donor Chelating Ligands

The C^N chelating agents deliver donor atoms, which stabilize the respective complexes and prevent their decomposition in physiological environments, modulating their catalytic and pharmacological activity.

Monochloridoiridium(III) complexes, as most Ir(III) complexes, were initially considered to be too inert to exhibit high antitumor activities. This is probably because hydrolysis is usually a key step in the modes of action of known metal-based antineoplastic agents, and, yet, the process of exchanging an aqua ligand on the hexaaqua cations [Ir(H_2_O)_6_]^3+^ takes a long time. In fact, it has been found that the replacement of three water molecules in [Ir(H_2_O)_6_]^3+^ by facial cyclopentadienyl ligands led to a dramatic increase in the lability of the remaining aqua ligands [60]. This labializing effect was less pronounced for neutral co-ligands such as benzene or p-cymene. Introduction of the 2,2′-bipyridyl ligand reduced the lability of the remaining aqua ligand in [(L)M(H_2_O)(bpy)]^2+^ by about two orders of magnitude down for the monochloridoiridium(III) complex, where L is Cp*. Polypyridyl complexes of the type [(η^5^-Cp*)MCl(pp)]^+^ also undergo rapid aquation due to the strong trans-effect of the anionic [Cp*]^−^ ligand. The equilibrium conditions for the monochloridoiridium(III) complexes were reached for less than 1 min in the case of 2,2′-bipyridine and 1,10-phenanthroline ligands [61]. The unsubstituted Cl^−^ species would probably prevail in extracellular fluids, but activation by Cl^−^/H_2_O exchange should be more favorable in the cell cytoplasm and cell nucleus.

**Figure 18:**
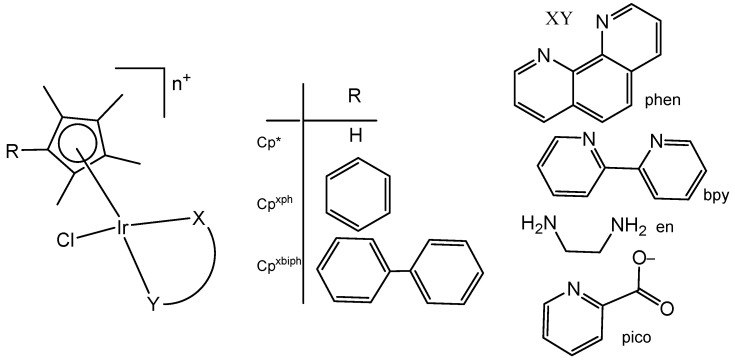
General structures of cytotoxic half-sandwich monochloridoiridium(III) complexes with substituted tetramethylcyclopentadienyl ligands, where n = 1, X = N; and n = 0, X = C, O.

Liu et al. have revealed that the cytotoxic activity can be improved for the monochloridoiridium complexes [(η^5^-C_5_Me_4_R)IrCl(pp)]^+^ (pp = en, bpy, phen) by including a phenyl (ph) or biphenyl (biph) substituent R into the cyclopentadienyl ring (Figure 18). The IC_50_ values for the bpy and phen complexes against A2780 cancer cells decreased strongly in the order Cp* > Cp^xph^ > Cp^xbiph^ [61]. While their Cp* complexes were both inactive with IC_50_ > 100 μM, the values of 15.86 μM and 6.70 μM have been recorded for phenyl-substituted and 0.57 μM and 0.72 μM for biphenyl-substituted bpy and phen complexes. The enhancement in activity along the series of C_5_Me_4_R co-ligands correlated with the increases in hydrophobicity, cellular uptake, and DNA intercalation by the phenyl- and biphenyl-substituents, as well as with the degrees of aquation for the 1,10-phenanthroline complexes. Enhanced hydrophobicity and the related higher intracellular levels of monochloridoiridium(III) could probably be the main reason for the improved activity. Aquation was rapid for the Cp*Ir(III) phen complex but slowed down on the inclusion of phenyl- and biphenyl-substituents into the Cp ring. These monochloridoiridium(III) complexes with N,N-chelating ligands were observed to bind quickly and to form adducts almost completely with 9-ethylguanine in competition studies with 9-ethyladenine [61]. The hydrophobicity and intercalative ability of the ligands Cp^xph^ and Cp^xbiph^ have made a major contribution to the antineoplastic potency of monochloridoiridium organometallic half-sandwich complexes [(η^5^-Cp^x^)Ir(L)Cl]^0/+^ of 1,10-phenanthroline, 2,2′-bipyridine, ethylenediamine and picolinate, where Cp^x^ is Cp*, tetramethyl(phenyl)cyclopentadienyl (Cp^xph^), or tetramethyl(biphenyl)cyclopentadienyl (Cp^xbiph^).

Replacement of the polypyridyl ligands (pp) by methyl-substituted derivatives, such as 4-Mephen, 5-Mephen, 4,7-Me_2_phen, 5,6-Me_2_phen and Me_2_dppz, has led to a significant improvement in their antiproliferative activity towards human MCF-7 and HT-29 cancer cells [62]. The monochloridoiridium complexes with the large dppz ligand exhibited closely similar IC_50_ values of 2.3 μM and 7.4 μM for MCF-7 and HT-29 cell lines, which were somewhat higher than those of 0.8 μM and 1.8 μM for the free ligand against the tested cell lines. The approximately four-fold higher level for the Cp*monochloridorhodium(III) dppz complex correlated with its increased antiproliferative activity, despite being markedly lower than that of its Cp*iridium(III) counterpart [63], for which the Cl^−^/H_2_O exchange to afford the less hydrophobic aqua dilation would be meaningly slower. In contrast to the more active Cp*monochloridorhodium(III) analogs, only modest IC_50_ values of 20–60 μM were recorded for the Cp*monochloridoiridium(III) complexes of 5-Clphen, 5-NO_2_phen and 5,6-Me_2_phen. The complex [(η^5^-Cp*)IrCl(phen)](CF_3_SO_3_) was practically inactive (IC_50_ > 100 μM) towards MCF-7 cells. The same was true for [(η^5^-Cp*)IrCl(pytz)]PF_6_, where pytz is 2-(pyridine-2-yl)thiazole, towards A2780 and cisplatin-resistant A2780cisR cells [64]. The preference of Cp*^−^monochloridoiridium(III) complexes to bind DNA depends on the aromatic surface area of the pp chelators, although DNA is known as a potential intracellular target for Ru(II)-arene antineoplastic complexes [65]. For instance, the interaction of [(η^5^-Cp*)IrCl(dppz)]^+^ with CT DNA highlighted the competitive nature of the intercalative and covalent Ir-N(nucleobase) binding modes for the soft Ir(III) atom. A time-dependent UV–Vis study at r = 0.1 revealed kinetically favored DNA intercalation at room temperature. Easy aquation has led to rapid formation of the dicationic aqua complex followed by the thermodynamically favored Ir-N(nucleobase) binding to DNA. The measurements were in agreement with covalent binding to DNA also for the monochloridoiridium(III) complexes of dipyrido[3,2-d:2′,3′-f]quinoxaline and (benzo[i]dipyrido[3,2-a:2′,3′-c]phenazine [63].

**Figure 19:**
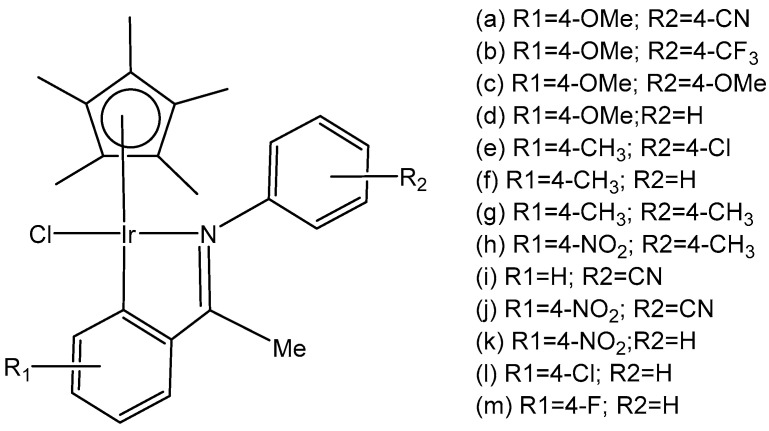
Half-sandwich Schiff base monochloridoiridium(III) complexes.

Mou et al. synthesized and investigated a series of half-sandwich Schiff base monochloridoiridium(III) complexes, Figure 19(a–m) [66]. All the complexes have shown higher potency than that of cisplatin in K562, K562/A02, MCF-7, MCF-7/ADM, and A549 cell lines. They demonstrated antiproliferative activities against the leukemia K562 cells with IC_50_ values of 0.26–4.77 μM. Studies have revealed that the compounds, containing stronger electron donating groups, showed greater potency than their counterparts without electro-donating groups. The monochloridoiridium compound, Figure 19(k), had the least activity (IC_50_ = 4.47) as it contained a strong electron-withdrawing nitro group while the compound, shown in Figure 19(c), containing two electro-donating methoxy groups had the highest antineoplastic activity (IC_50_ = 0.26 µM), better than cisplatin in K562, MCF-7, MCF-7/ADM. Mechanistic studies have illustrated that the monochloridoiridium complexes (Figure 19) increased the level of reactive oxygen species and induced the apoptosis of K562 cells, mediated by the intrinsic mitochondria apoptotic pathway.

**Figure 20:**
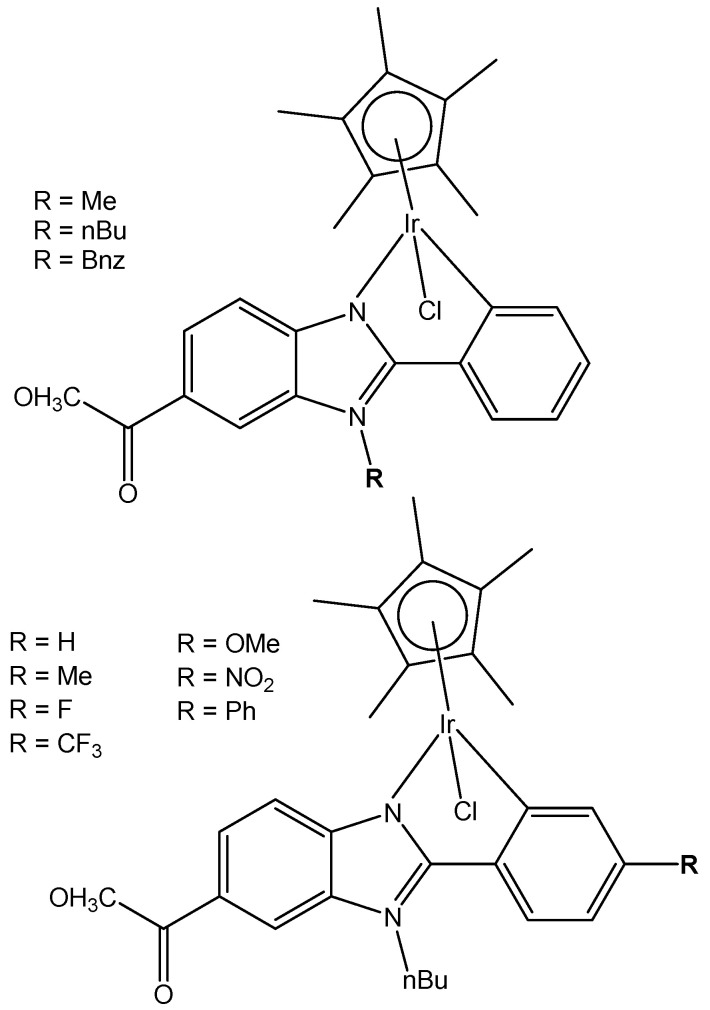
Monochloridoiridium(III) compounds with cyclometalated benzimidazole ligands.

A series of N^C cyclometalated monochloridoiridium(III) compounds of the type [(η^5^-C_5_Me_5_)IrCl(κ^2^-*N*,*C*-L)], where HL is methyl 1-butyl-2-arylbenzimidazolecarboxylate with varying substituents (H, Me, F, CF_3_, MeO, NO_2_, and Ph) in the R_4_ position of the phenyl ring of 2-phenylbenzimidazole chelating ligand of the monochloridoiridium complexes (Figure 20), have been described [67]. The complexes were synthesized by using the 1-butyl-2-phenyl-1H-benzo[d]imidazole-5-carboxylate of methyl with different substituents at the C4 position of the phenyl ring as ligands. The cytotoxic activity of the new monochloridoiridium(III) compounds has been evaluated in a panel of cell lines (A2780, A2780cisR, A427, 5637, LCLC, SISO, and HT29) in order to investigate their structure–activity relationships. Cytotoxicity studies have shown that the methyl substituent (R = Me) has decreased the cytotoxic effect in most of the tested cell lines. The presence of phenyl (R = Ph) has enhanced the cytotoxicity with reference to cisplatin, except in the LCLC and SISO cell lines. The monochloridoiridium(III) complex without substituents in the benzimidazole ligand has shown good results in A-2780 and cisplatin resistant A2780/cisR cell lines. All the studied monochloridoiridium(III) compounds exceeded acquired resistance to cisplatin. The new compounds increased caspase-3 activity and the production of ROS in A2780 cells.

**Figure 21:**
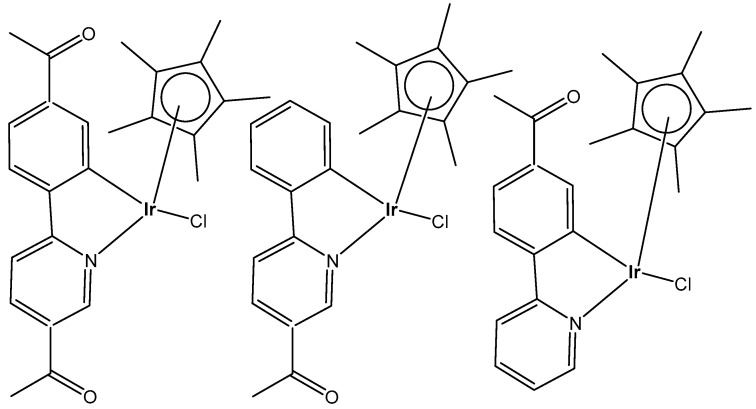
Structures of monochloridoiridium(III) piano-stool complexes.

Wootton et al. synthesized novel organo-monochloridoiridium(III) anticancer complexes (Figure 21), each having reactive sites both at the metal and ligand [68]. The authors characterized unusual adducts from reactions between the novel Ir(III) complexes, each possessing reactive sites both at the metal (coordination by substitution of a labile chlorido ligand) and on the ligand (covalent bond formation involving imine formation by one or two aldehyde functions). To understand the mode of action, it was important to discover how the monochloridoiridium(III) complexes bind to biomolecules. It was found that the aldehyde functionalized iridium piano-stool Cp* compounds bonded to peptides via a coordination bond between the iridium center and histidine residues available. These complexes have been shown to bind to lysine residues within biomolecules via condensation reactions, creating a covalent imine linkage.

**Figure 22:**
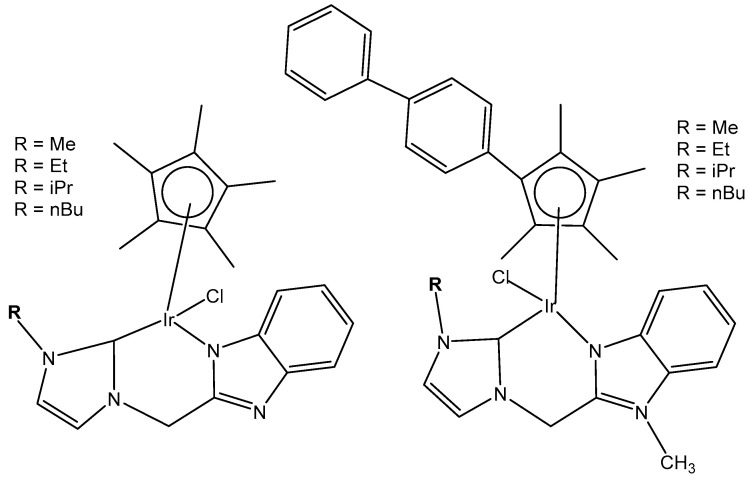
Monochloridoiridium(III) complexes with 1-methyl-3-((1-methyl-1H-benzo[d]imidazol-2-yl)methyl)-2,3-dihydro-1Himidazole-2-ide ligand.

A series of half-sandwich iridium(III) antitumor complexes [(η^5^-Cp^x^)Ir(C^N)X]Y with benzimidazole-appended imidazolium-based *N*-heterocyclic carbene (NHC) ligands, where Cp^x^ is pentamethylcyclopentadienyl or its biphenyl derivative (Cp^xbiph^), X is a leaving group, Y is a counter-ion and C^N is a NHC chelating ligand, have been successfully synthesized and characterized [69]. The most important factors to be considered in the rational design are the electron-donating capacity of the arene ligands, the exchange of the leaving groups, as well as the type and size of the counterions. The C^N ligand used for the synthesis of monochloridoiridium(III) complexes was 1-methyl-3-((1-methyl-1H-benzo[d]imidazol-2-yl)methyl)-2,3-dihydro-1H-imidazole-2-ide. The arenes used to modulate the properties were pentamethylcyclopentadiene and [(1,10-biphenyl)-4-yl]-tetramethyl-cyclopentadiene. The obtained half-sandwich monochloridoiridium complexes (Figure 22) have demonstrated excellent antineoplastic activity against the tested A-549 and BEAS-2B cell lines with improved effects than that reported for the classical drug cisplatin. The Ir(III) complexes with the largest arene ligand (nBu) have displayed better activity, which was associated with the hydrophobic character of the arene helping it to enter the tumor cells. The activity trend was found to be in the following order: nBu > iPr > Et > Me. The observed results have suggested that the antitumor activity can be controlled by modifying the C^N ligands and arenes. The monochloridoiridium(III) complexes of this series could bonded to BSA by a static quenching mode, catalyzing the change of NADH to NAD**^+^** and inducing the production of ROS, playing an imperative role in regulating cell apoptosis. Confocal microscopy studies have shown that monochloridoiridium(III) complexes could specifically target lysosomes in cells, followed an energy-dependent cellular uptake mechanism and damaged the integrity of lysosomes. The studied complexes have also caused a marked loss of mitochondrial membrane potential.

**Figure 23:**
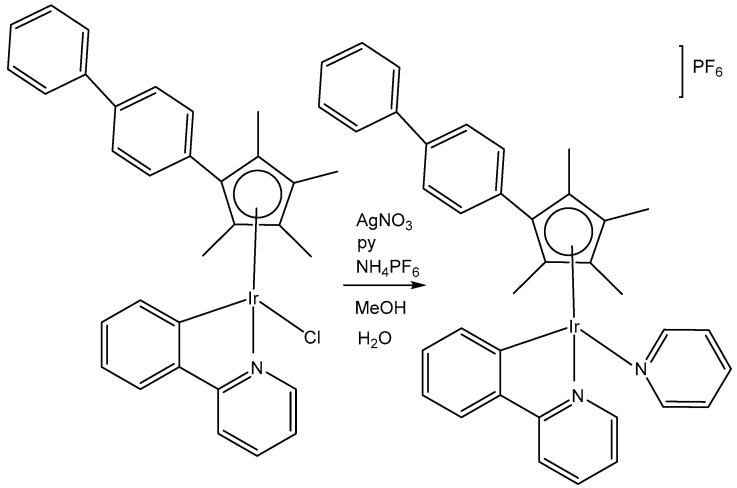
Synthetic route for the iridium(III) pyridine complex [(η^5^-Cp^xbiph^)Ir(phpy)py]PF_6_.

The organoiridium(III) complex [(η^5^-Cp^xbiph^)Ir(phpy)(Cl)], which contains π-bonded biphenyltetramethylcyclopentadienyl (Cp^xbiph^) and C^N-chelated phenylpyridine (phpy) ligands, undergoes the rapid hydrolysis of a Cl^−^ ligand. In contrast, the pyridine complex [(η^5^-Cp^xbiph^)Ir(phpy)py]^+^ hydrolyzes slowly and has been found to be more potent (in nanomolar amounts) than [(η^5^-Cp^xbiph^)Ir(phpy)(Cl)] and cisplatin against a wide range of tumor cells. The comparison of the hydrolysis of the monochloridoiridium(III) complex [(η^5^-Cp^xbiph^)Ir(phpy)(Cl)] with that of the pyridine complex [(η^5^-Cp^xbiph^)Ir(phpy)py]^+^ (Figure 23) was reported [70]. It has been shown that the monodentate pyridine ligand had a major influence on the chemical reactivity and antineoplastic potency. The pyridine ligand protected the complex from a fast reaction with intracellular glutathione. The high potency of the pyridine complex [(η^5^-Cp^xbiph^)Ir(phpy)py]^+^ correlated with its ability to considerably increase the ROS level in cancer cells.

The cytotoxic activity of [(η^5^-Cp^xbiph^)Ir(phpy)(Cl)] and [(η^5^-Cp^xbiph^)Ir(phpy)py]^+^ towards different cancer cells and their selectivity over normal cells were investigated [70]. The complex [(η^5^-Cp^xbiph^)Ir(phpy)py]PF_6_ has displayed high potency and nanomolar activity against human ovarian A2780 cancer cells. It was approximately six times more potent than [(η^5^-Cp^xbiph^)Ir(phpy)(Cl)] and ten times more active than the clinically used cisplatin. Moreover, it was considerably less toxic against the normal human lung fibroblast MRC-5 cells than against cancer cells. It has been proven that the studied complex disturbed cellular redox homeostasis. The cellular accumulation of iridium(III) and the production of ROS in cells have been related to the oxidation–reduction chemistry of the tested complexes. The unprecedented ability of these iridium complexes to generate H_2_O_2_ by catalytic hydride transfer from the coenzyme NADH to O_2_ has been observed. Most probably, the ability of the cyclopentadienyl iridium(III) complexes to accept a hydride from the NADH coenzyme can be linked to ROS generation. It has been demonstrated that iridium(III) organometallic complexes can be used as highly effective oxidants for the treatment of cancer, proven by the achieved facile conversion of coenzyme NADH to NAD^+^ through hydride transfer using this type of Ir(III) complex. Hydride transfer from NADH resulted in the formation of Ir–H species. The hydride could further be transferred to oxygen to generate H_2_O_2_. These organoiridium complexes would provide a means of probing NADH-mediated cell signaling pathways and coupling hydrogenations to biological processes. The complex [(η^5^-Cp^xbiph^)Ir(phpy)py]PF_6_ was an example of an antineoplastic complex, capable of generating significant ROS levels in cancer cells, playing an important role in the observed antineoplastic activity. Notably, such organoiridium complexes could be presented as promising anticancer candidates with new modes of action, different from that of platinum drugs. This novel strategy for the rational design of oxidant catalytic organoiridium drugs, involving catalytic hydride transfer from the coenzyme NADH to oxygen to produce H_2_O_2_ as a product, might be highly effective for the therapy of Pt-resistant tumors and for effective oxidant therapy.

**Figure 24:**
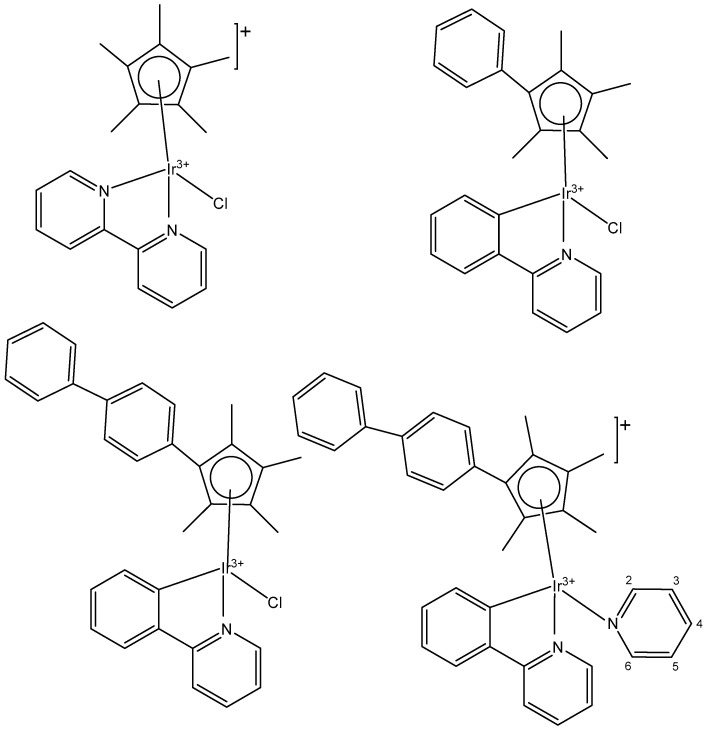
Half-sandwich organometallic Ir(III) complexes of the type [(η^5^-Cp^x^)Ir(LL)Z]^0/n+^, where Cp^x^ = Cp*, Cp^xph^ ((tetramethyl-(phenyl)cyclopentadienyl) or Cp^xbiph^ ((tetramethyl(biphenyl)-cyclopentadienyl).

A variety of organometallic Ir(III) cyclopentadienyl complexes of the type [(η^5^-Cp^x^)Ir(LL)Z]^0/n+^, where Cp^x^ = Cp*, Cp^xph^ ((tetramethyl-(phenyl)cyclopentadienyl) or Cp^xbiph^ ((tetramethyl(biphenyl)-cyclopentadienyl), LL = bidentate ligand with N, O or C donor atoms, Z = Cl, H_2_O or pyridine (py), have been synthesized and characterized as potential antineoplastic agents (Figure 24) [71]. There are effective strategies to achieve the potent anticancer activity, involving modifications of the ligands around the Ir(III) center. It has been found that the antiproliferative activity decreased markedly from inactive to highly potent when phenyl or biphenyl was introduced in place of the methyl group on the Cp ring. The anticancer activity was improved significantly by the replacement of neutral N^N-chelating ligands with negatively charged C^N-chelating ligands. The monodentate ligand Z (Cl^−^) was readily substituted by H_2_O because of hydrolysis, followed by interaction with biomolecules. The further addition of biphenyl substituents and the replacement of Cl^−^ by pyridine led to nanomolar activity. These derivatives were much more active than cisplatin.

**Figure 25:**
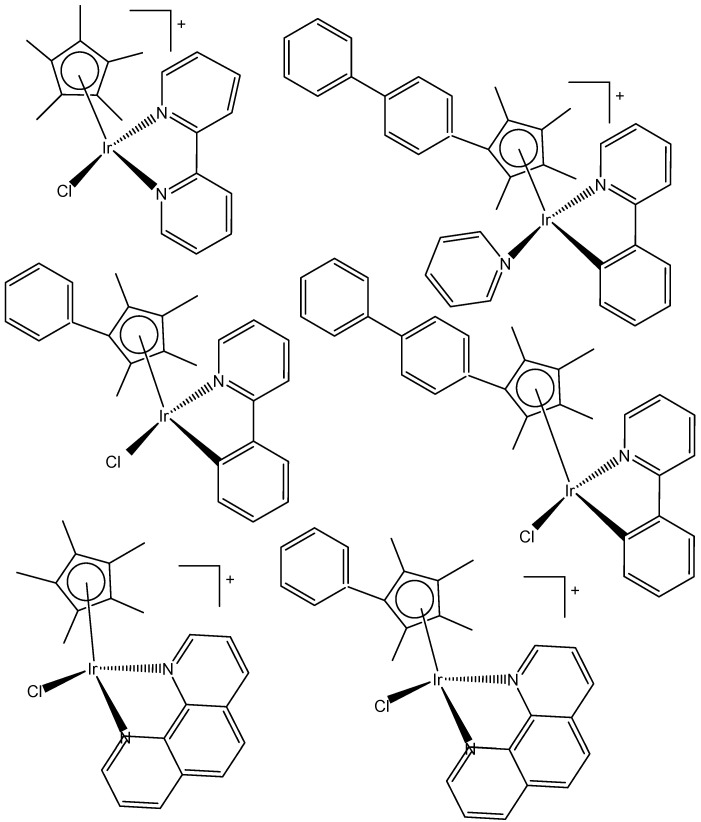
Ir(III)-Cp* complexes with K^2^N,C ligands.

The dependency of cytotoxic activity and other biological properties on the size of the Cp^x^ ring in monochloridoiridium complexes was discussed by Liu et al. [61]. Particularly, inactive Ir(III)-Cp* complexes could be turned into active compounds when their Cp* ring was replaced by more extended rings, viz., 2,3,4,5-tetramethyl-1-phenylcyclopenta-2,4-dienyl (Cp^ph^) or 1-(biphenyl-4-yl)-2,3,4,5-tetramethylcyclopenta-2,4-dienyl (Cp^bph^). This was observed especially for 2,2′-bipyridyl (bpy) and 1,10-phenanthroline (phen) complexes. The Cp^bph^ complexes have reached even submicromolar activity. The activity improvement with increasing phenyl substitution on the Cp^x^ ring was confirmed by many researchers for various iridium complexes [72,73]. Organometallic monochloridoiridium(III) cyclopentadienyl complexes [(η^5^-Cp*)Ir(C^N)Cl] of the C^N ligands 2-(*p*-tolyl)pyridine, 2-phenylquinoline, 2-(2,4-difluorophenyl)pyridine for Cp* = Cp^x^, C^N = 2-phenylpyridine for Cp* = tetramethyl(phenyl)cyclopentadienyl (Cp^xph^), and C^N = 2-phenylpyridine for Cp* = tetramethyl(biphenyl)cyclopentadienyl (Cp^xbiph^) (Figure 25) have been synthesized and characterized [73]. All these complexes have hydrolyzed rapidly in a water solution. It has been found that the complexes bonded strongly to 9-ethylguanine (9-EtG) and moderately to 9-methyladenine (9-MeA). All the complexes have shown effective cytotoxicity, with IC_50_ values ranging from 6.5 to 0.7 μM against human ovarian A2780 cancer cells. Their activity has been improved with additional phenyl substitution on the Cp* ring: Cp^xbiph^ > Cp^xph^ > Cp^x^. Cp^xbiph^. In numerous subsequent studies, a Cp^x^ ring extension was successfully applied for the improvement of the antiproliferative activity of parent Cp* complexes. Nevertheless, this dependence should not be accepted as straightforward, because, in numerous cases, it was Cp* complexes that outperformed their Cp^ph^ and Cp^bph^ analogs. In different cases, it was found that the Cp*-extension was not required in the case of N,C ligands, signifying a different mode of action. It was demonstrated that Cp*-Ir(III) compounds have had a redox mechanism of action [74,75].

The potency of the Ir(III)-Cp* complexes could be further improved by employing the isoelectronic K^2^N,C phpy^−^ ligand and its derivatives [72,73] instead of phen, Figure 25. Replacing the N,N-chelating ligand 2,2′-bipyridine (bpy) in the monochloridoiridium(III) pentamethylcyclopentadienyl (Cp*) complex [(η^5^-C_5_Me_5_)Ir(bpy)Cl]^+^ with the C,N-chelating ligand 2-phenylpyridine (phpy) to give [(η^5^-C_5_Me_5_)Ir(phpy)Cl] has improved the cytotoxicity toward human ovarian A2780 cancer cells (IC_50_ = 10.8 μM for [(η^5^-C_5_Me_5_)Ir(phpy)Cl]) [72]. Ir–Cl hydrolysis was rapid for both complexes. The complex [(η^5^-C_5_Me_5_)Ir(phpy)Cl] formed adducts preferentially with 9-ethylguanine (9-EtG) rather than with 9-methyladenine (9-MeA). The X-ray crystal structure of [(η^5^-C_5_Me_5_)Ir(phpy)(9-EtG-*N*7)]NO_3_·1.5CH_2_Cl_2_ confirmed the N7 binding to guanine. The strong nucleobase binding and high hydrophobicity of the monochloridoiridium complex [(η^5^-C_5_Me_5_)Ir(phpy)Cl] have probably contributed to its significant antineoplastic activity. The introduction of phpy^−^ has also led to improved effectiveness (IC_50_ = 2.14 μM) for the Cp^xph^ complex in comparison to its phen analogs. As indicated by its logP values for [(η^5^-Cp*)IrCl(phen)]^+^, the overall charge reduction from +1 to 0 significantly increased the hydrophobicity of the monochloridoiridium complex [(η^5^-Cp*)IrCl(phpy)] [72]. The associated higher level of cellular uptake was presumably the major cause for the improved cytotoxic activity of the complexes with phpy^−^ and its derivatives dfphpy^−^, mphpy^−^ and phq^−^. As in the case of the pp complexes, a strong binding of the K^2^N,C Cp*Ir(III) complexes to guanine N7 atoms might also contribute to their relatively high activity [73]. These data have demonstrated how the aqueous chemistry, nucleobase binding, and anticancer activity of C,N-bound monochloridoiridium(III) cyclopentadienyl complexes could be controlled and fine-tuned by the modification of the chelating and cyclopentadienyl ligands.

**Figure 26:**
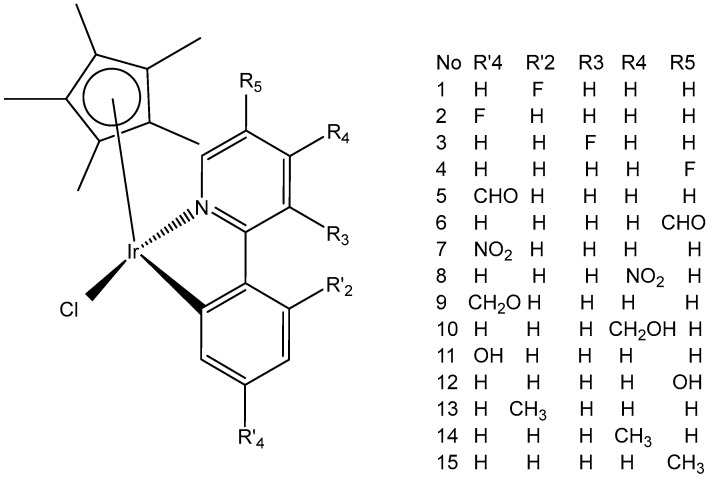
Complexes of the type [(η^5^-Cp*)Ir(2-(R′-phenyl)-R-pyridine)Cl] with different substituents.

Millett et al. have investigated the effects of electron donating and electron-withdrawing substituents on *C,N*-chelating 2-PhPy ligands on the physicochemical and biological activity of their monochloridoiridium(III) complexes [76]. This comprehensive study has shown that small changes in the functionalization of the chelating ligands in half-sandwich monochloridoiridium(III) complexes could lead to profound effects on the bioactivity of Ir(III) complexes. The synthesis, characterization, and cytotoxic activity of fifteen monochloridoiridium(III) half-sandwich complexes of the type [(η^5^-Cp*)Ir(2-(R′-phenyl)-R-pyridine)Cl] with electron-donating (–OH, –CH_2_OH, –CH_3_) or electron-withdrawing (–F, –CHO, –NO_2_) groups at various places on the 2-phenylpyridine (2-PhPy) have been reported. Hydrolysis of the studied complexes was rapid. The monochloridoiridium(III) complexes have shown preferential binding to the nucleobase 9-ethylguanine over 9-methyladenine and were active catalysts for NADH to NAD^+^ cellular oxidation, with an enhanced catalytic ability of hydroxyl substituents compared to fluorine. The antiproliferative activity trials in A2780, MCF-7, A549, and HCT116 tumor cells showed IC_50_ values ranging from 1 to 89 μM. The most active complex, [(η^5^-Cp*)Ir(2-(2′-methylphenyl)pyridine)Cl] (Figure 26, No13) with IC_50_ = 1.18 μM against A2780 was 10 times more active than the parent compound [(η^5^-Cp*)Ir(2-phenylpyridine)Cl]. A growth in hydrophobicity led to enhanced cellular iridium accumulation in A2780 ovarian cells and better activity. Notably, this work has also highlighted that the inclusion of substituents of different natures and positions in 2-PhPy ligands could provide monochloridoiridium complexes with enhanced potency without the use of extended cyclopentadienyl ligands, a strategy that might be beneficial for the development of this class of fine-tuned complexes.

### 5.2. Monochloridoiridium(III) Cyclopentadienyl Complexes with N,N-Donor Chelating Ligands

The organo-iridium anticancer complexes of the type [(Cp^X^)Ir(N^N)Cl]^+^ (Cp^X^ = Cp*, Cp^xph^, or Cp^xbiph^) with various N^N chelating ligands and chloride ions as a monodentate ligand (some of them shown in Figure 25) are the most popular in this class of compounds [72,73]. These low-spin 5d^6^ iridium complexes have been found to be highly unstable toward aquation but usually inactive as antitumor agents. The adding of one or two phenyl rings to the Cp* has improved the potency of these complexes towards cancer cells, binding directly to DNA (particularly N7 of G). As a whole, the majority of half-sandwich monochloridoiridium complexes with N^N coordinating ligands typically undergo fast hydrolysis and can readily form adducts with 9-ethylguanine (9-EtG).

**Figure 27:**
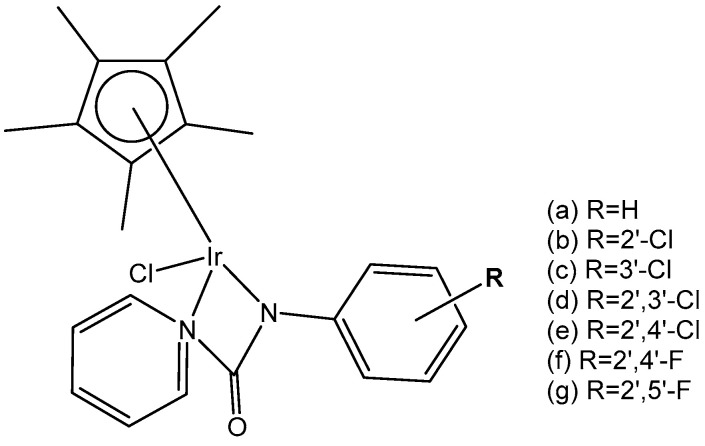
Structure of iridium complexes with (N,N)-bound picolinamide ligands.

Eight monochloridoiridium complexes, Figure 27(a–g), based on iridium(III) with (*N,N*)-bound picolinamide ligands have been prepared for use as anticancer agents by Almodares et al. [77]. The complexes have shown promising cytotoxicity, with the presence, position, and number of halides having a significant effect on the corresponding IC_50_ values. They have been tested on A2780 cells for 5 days of exposure. The monochloridoiridium complex, Figure 27(a), has shown poor activity with an IC_50_ value of 66 µM; however, the addition of a chloride ion to the ligand, Figure 27(b,c), showed increased activity (IC_50_ = 25–33 µM). The compounds, Figure 27(d,e), have demonstrated even higher cytotoxic activity with IC_50_ values of 19 µM and 23 µM, respectively. It has been shown that a chloride ion in the ortho-position of arene was more efficient compared to that in a meta-position. Similar trends were observed in diflouro complexes although they were less potent compared to chloro-analogs. Further studies on the mechanism of action have indicated that Trx-R inhibition could be the possible action mechanism of the studied monochloridoiridium complexes (Figure 27) [77].

**Figure 28:**
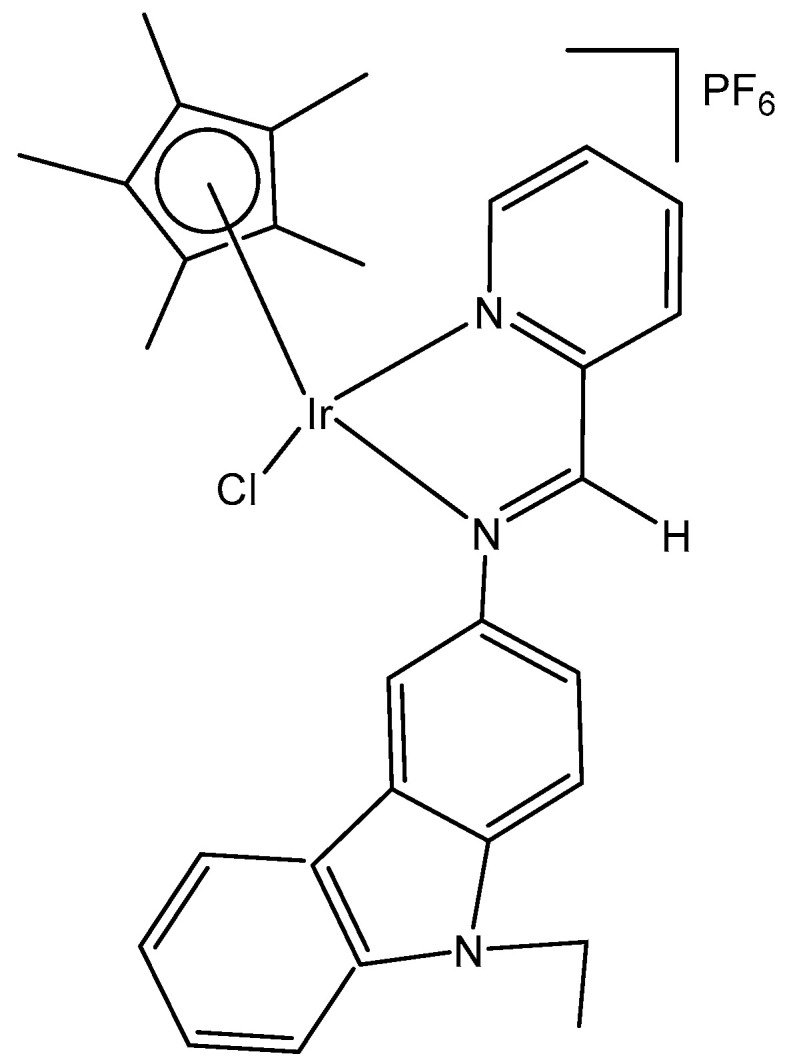
Structure of monochloridoiridium complex [(η^5^-C_5_Me_5_)IrCl(L)]PF_6_ of 9-ethyl-N-(pyridine-2-yl methylene)-9H-carbazole-3-amine.

Thangavel et al. have synthesized and characterized a half-sandwich monochloridoiridium complex of a biologically active carbazole N,N′ ligand [(η^5^-C_5_Me_5_)IrCl(L)]PF_6_ (Figure 28), where L = 9-ethyl-N-(pyridine-2-yl methylene)-9H-carbazole-3-amine) [78]. The obtained iridium complex had a pseudo-octahedral “piano-stool” geometry. The monochloridoiridium complex showed good cytotoxic activity compared to cisplatin in MCF-7 breast cancer cells, which may be attributed to the hydrophobicity of η^5^-C_5_Me_5_, which was higher than that of the benzene ring. Cell morphology, apoptosis, cell cycle arrest and molecular docking have also been studied. The monochloridoiridium(III) complex was able to arrest the S cell cycle, and further studies revealed that the complex bonded in the active site of COX-2 via H-bonding. Fluorescence studies have exposed that the complex had a good affinity to interact with biomolecules like DNA, proteins and RNA, which might be responsible for inducing apoptosis or causing cell cycle inhibition in MCF-7 cancer cells (Figure 28) [78].

**Figure 29:**
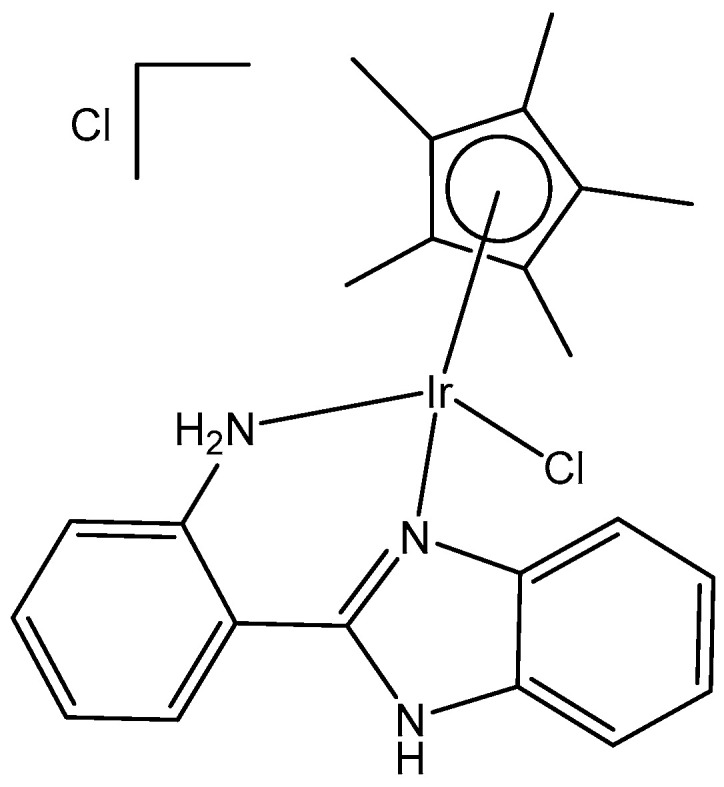
Iridium(III)) complex [(arene)Ir(III)Cl(N^N)]Cl with 2-(1*H*-benzo[*d*]imidazol-2-yl)aniline.

The anticancer activity and the binding to DNA and proteins of the cationic iridium(III) complex [(arene)Ir(III)Cl(N^N)]Cl (Figure 29) were reported by Kumar et al. [79]. The obtained iridium(III) complex has been synthesized by employing the bidentate ligand 2-(1*H*-benzo[*d*]imidazol-2-yl)aniline, and its antineoplastic activity against the cervical cancer SiHa cell line has been investigated. The Ir(III) complex has exhibited excellent cytotoxicity and in vitro antineoplastic activity on the SiHa cell proliferation (IC_50_ = 6.14 μM), while the effect of the free ligand has been negligeable. This monochlorido-substituted iridium complex has penetrated tumor cells binding DNA effectively. The strong binding with calf thymus DNA (CT-DNA) and bovine serum albumin (BSA), promoted by the studied compound, can be explained by the strong electrostatic and H-bonding interactions. These results have been supported by DFT calculations and molecular docking studies.

**Figure 30:**
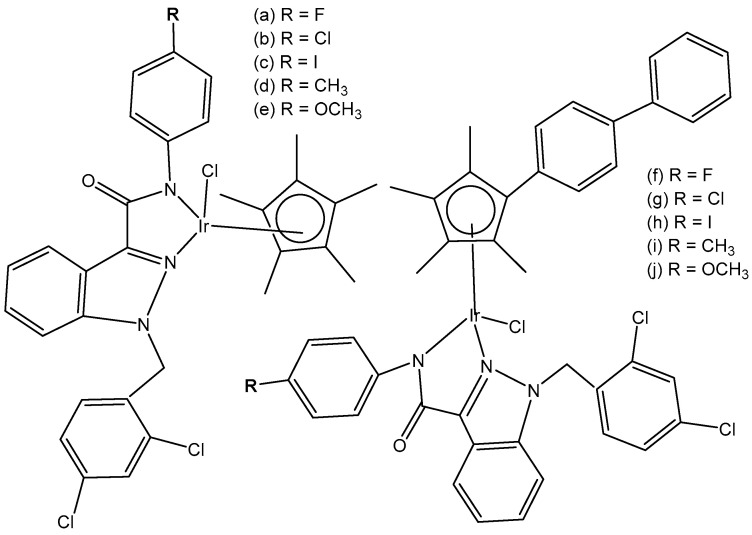
Structures of monochloridoiridium(III) complexes containing lonidamine amide derivatives.

Xie et al. have synthesized and characterized half-sandwich monochloridoiridium complexes, containing lonidamine amide derivatives, Figure 30(a–j) [37]. Contrasting lonidamine, which acts on mitochondria-targeting tumors exclusively through the glycolytic pathway, its monochloridoiridium(III) complexes successfully targeted lysosomes and induced lysosomal damage. Antiproliferation studies have shown that most Ir(III) complexes had better antitumor activity against A549 and HeLa cells compared to that of cisplatin. The antitumor tests of ligand derivatives have proven that they exhibited excellent antitumor activity only after binding to the metal center. It has been found that the antineoplastic activity depended on the substituents of the ligands. The fluorine-containing complex, Figure 30(f), has shown the best inhibitory effect on the proliferation of A549 and HeLa cells, having higher antineoplastic activity of about 2.5 times of that of cisplatin. Similarly, the complex, shown in Figure 30(j), displayed 2.2 times better activity compared to cisplatin. Against the HeLa cell line, monochloridoiridium(III) complexes, Figure 30(e,f,h,i), exhibited superior activity. The bovine serum albumin (BSA) binding test has shown that the complexes had the ability to bind to BSA. Complexes, shown in Figure 30(e,f), interacted with BSA in the static quenching mechanism, confirming the binding ability. The studied monochloridoiridium complexes could also cause changes in mitochondrial membrane potential and could influence the ROS generation better than the active control. NADH/NAD^+^ transformation experiments have been used to determine if the production of ROS was caused by the transformation of NADH/NAD^+^. The authors have also discovered the way that the complexes enter cells. The complex, Figure 30(e), showed specific targeting towards lysosomes and induced apoptosis [37].

**Figure 31:**
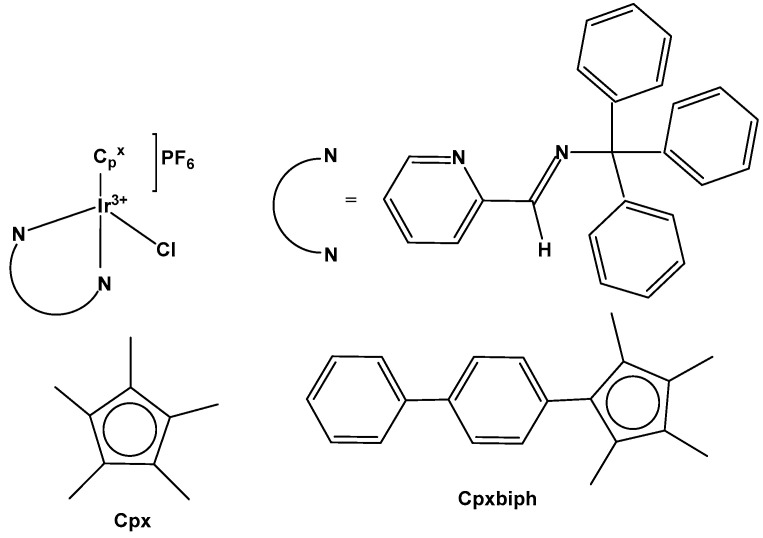
Structure of monochloridoiridium(III) complexes with general formula [(η^5^-Cpx)Ir(N^N)Cl]PF_6_.

Half-sandwich iridium-based anticancer complexes commonly suffer from uncertain targets and mechanisms of action. Li et al. have reported the observation of the images of half-sandwich monochloridoiridium complexes [(η^5^-Cpx)Ir(N^N)Cl]PF_6_, Figure 31, in cells, detected by confocal microscopy [38]. Confocal microscopy provided insights to microscopic mechanisms including cellular uptake, distribution, and interaction with biological targets. The monochloridoiridium(III) complexes have exhibited high antitumor activity, around 4.2 times superior than that of cisplatin towards A549 lung cancer cells. The study has shown that the cyclopentadienyl monochloridoiridium complex mainly accumulated in nuclei in A549 cells through energy-dependent active transport. DNA-binding studies involved interaction with the nucleobases 9-ethylguanine, 9-methyladenine, ctDNA, and plasmid DNA. The determination of BSA binding has shown that the complexes affected the tryptophan microenvironment pointedly but did not affect the tyrosine microenvironment throughout the binding process. Hydrolysis, stability, nucleobase binding, and catalytic NAD^+^/NADH hydride transfer tests for the studied monochloridoiridium complexes have also been performed. The complexes have caused apoptosis through the ROS-dependent mitochondrial pathway (Figure 31) [38].

**Figure 32:**
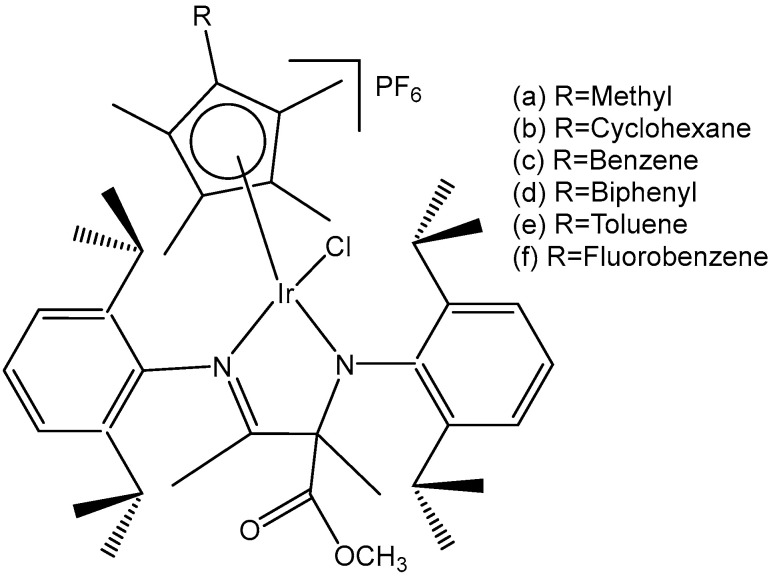
Structures of the iridium complexes [(η^5^-C_5_Me_5_)Ir(XY)Cl]^0/+^.

Organometallic six-coordinated half-sandwich monochloridoiridium(III) complexes, Figure 32(a–f), of the type [(η^5^-C_5_Me_5_)Ir(XY)Cl]^0/+^, where XY = N,N-chelated α-keto-β-diimine bidentate ligands, have been synthesized [80]. The complexes had shown sufficient stability in a DMSO–water solution. The cytotoxic activity for these monochloridoiridium(III) complexes has been examined by an MTT assay against A549, HeLa, and HepG2 cancer cells. All the complexes were found very effective against the treated cell lines with IC_50_ values ranging from 2.2 to 14.5 µM, which were less than that of the clinical drug cisplatin towards A549 and HeLa cancer cells. The complex, Figure 32(f), has been found to be the most potent complex against the tested cell lines, signifying that the activity could be enhanced by using fluorinated substituents in the η^5^-Cp* ring. Additional studies have displayed that the complexes exerted their antitumor efficacy by apoptosis or necrosis induction and by increasing the level of intracellular ROS. The investigation of the mechanism has shown that the most active monochloridoiridium complexes entered the cancer cells through an energy-independent pathway, and they were primarily found to be localized in the mitochondria and lysosomes of the cells. Notably, no selectivity was observed between tumor and normal cells with these complexes (Figure 32) [80].

**Figure 33:**
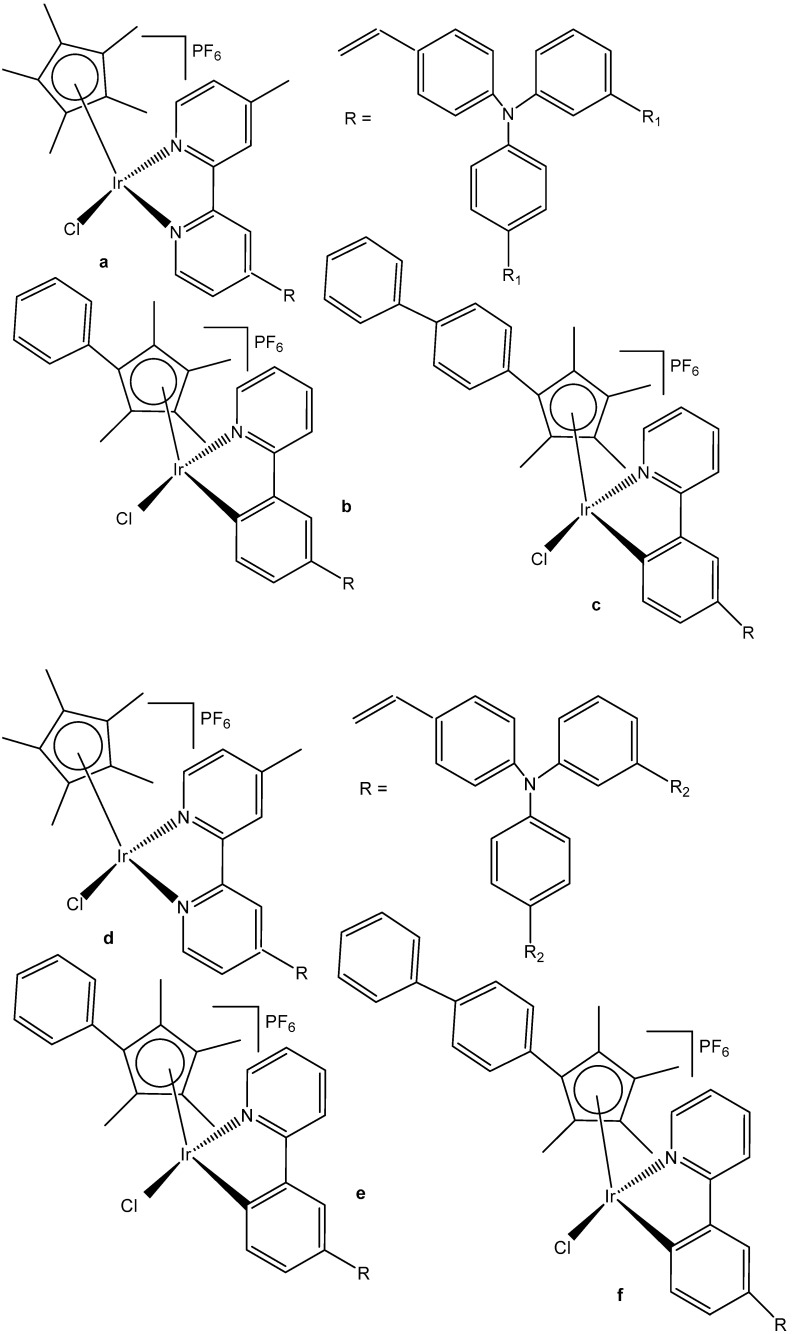
Structures of monochloridoiridium(III) complexes of the type [(η^5^-Cp^x^)Ir(N^N)Cl]PF_6_ with different TPA-substituted bipyridyl ligand groups, where R_1_ = CH_3_ (**a**,**b**,**c**) and R_2_ = OCH_3_ (**d**,**e**,**f**).

Organometallic half-sandwich monochloridoiridium(III) complexes of the type [(η^5^-Cp^x^)Ir(N^N)Cl]PF_6_, where Cp^x^ = Cp* or its phenyl (Cp^xph^) or biphenyl (Cp^xbiph^) derivatives, N^N = triphenylamine (TPA)-substituted bipyridyl ligand groups, Figure 33a–f, have been synthesized and characterized [81]. The antitumor activity of these complexes has been evaluated against human lung A549 cancer cells after 24 h of drug exposure. The results have shown that the monochloridoiridium complexes had better antitumor activity compared to cisplatin against A549 cells. Complexes, shown in Figure 33b,c, have displayed superior antiproliferative activity compared to other complexes, Figure 33a,d, due to enhanced lipid solubility caused by phenyl rings. However, the introduction of more phenyl rings, as seen in complexes in Figure 33c,f, has led to a decrease in antiproliferative activity due to the higher hydrophobicity of the chelating ligand. The monochloridoiridium complexes, presented in Figure 33a,d, have shown the best antineoplastic activity. The IC_50_ values for these compounds have varied from 1.5 to 7.1 µM, and they were up to 14 times more potent than cisplatin under the same conditions. However, no selectivity has been observed against the normal cell line BEAS-2B [81]. The monochloridoiridium(III) complexes (Figure 33) have exhibited excellent BSA and DNA-binding properties and a good ability to oxidize NADH to NAD^+^. The complexes induced apoptosis effectively and led to the emergence of ROS in cells.

**Figure 34:**
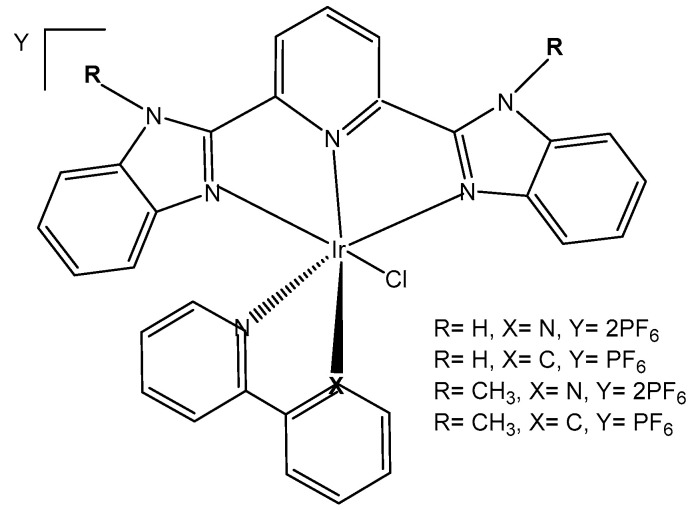
Monochloridoiridium(III) complexes with functionalized 2,6-bis(1*H*-benzo[*d*]imidazol2-yl)pyridine.

Cationic phosphorescent monochloridoiridium(III) complexes with the auxiliary ligands 2,2′-bipyridine (X = N) or 2-phenyl-pyridine (X = C), which allowed regulating the lipophilicity, Figure 34, have been synthesized and characterized [82]. The mixed-ligand phosphorescent monochloridoiridium complexes ([Ir(L_1_)(bpy)Cl](PF_6_)_2_, [Ir(L_1_)(ppy)Cl](PF_6_), [Ir(L_2_)(bpy)Cl](PF_6_)_2_, [Ir(L_2_)(ppy)Cl](PF_6_), bearing the ligands L_1_ = 2,6-bis(2-benzimidazolyl)pyridine; bpy = 2,2′-bipyridine; L_2_ = 2,6-bis(1-methyl-benzimidazol-2-yl)pyridine; ppy = 2-phenylpyridine, have displayed high luminescence quantum yields and long phosphorescence lifetimes. The widely utilized tridentate ligand, namely, N-functionalized 2,6-bis(1*H*-benzo[*d*]imidazol-2-yl)pyridine, has been used for the synthesis of these complexes. The cytotoxicity and photodynamic therapy activity of the studied monochloridoiridium complexes have been estimated against A-549, HeLa, MCF-7 and A-549-R cancer cell lines, as well as against normal LO2 cells and compared with the results of the control drug cisplatin. These compounds have displayed some selectivity towards human tumor cells over healthy cells. The best phototoxicity corresponded to the 2-phenyl-pyridine derivatives (X = C), primarily in the A-549 and A-549-R cells. Both 2-phenyl-pyridine-substituted compounds have shown phototoxicity between two to five times lower in normal LO2 cells than in cancer A-549 and A-549-R cells; therefore, these complexes could avoid the resistance to cisplatin when used as an approach in photodynamic therapy (PDT). It has been found that, when incorporating 2-phenyl-pyridine, the cellular uptake level and PDT efficiency increased, which has not been observed with the 2,2′-bipyridine derivatives. Concerning their luminescence properties, only 2-phenyl-pyridine-substituted derivatives have shown a distinct red fluorescence in lysosomes.

**Figure 35:**
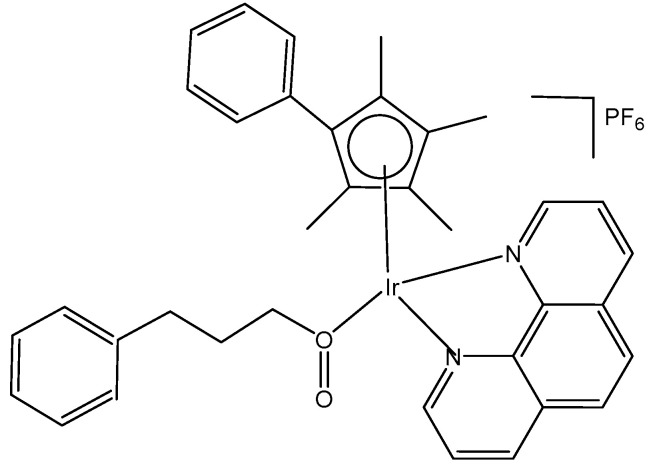
Half-sandwich Ir(III) complex [Ir(η^5^-Cpph)(phen)(pb)]PF_6_.

Similar to the above-mentioned complexes, Štarha et al. have recently reported a cytotoxic half-sandwich Ir(III) complex with a formula [Ir(η^5^-Cpph)(phen)(pb)]PF_6_ (Figure 35) containing a mono-dentate O-donor 4-phenylbutyrato ligand [83]. The complex cytotoxicity was found to be higher than that of conventional cisplatin (IC_50_ = 13 µM), and, also, it effectively inhibited HDAC activity (Figure 35) [83].

### 5.3. Monochloridoiridium(III) Cyclopentadienyl Complexes with N,O-Donor Chelating Ligands

**Figure 36:**
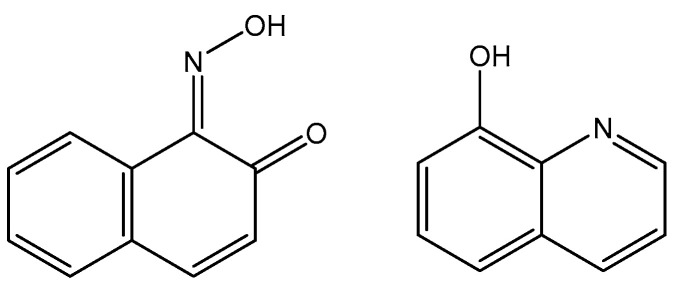
Structures of 1, 2-naphthoquinone-1-oximate and quinolin-8-ol ligands.

Examinations of half-sandwich Ir(III) complexes containing facial co-ligands have been provoked by the prosperous field of antitumor and antimetastatic water-soluble arene ruthenium(II) complexes of the corresponding types [(η^6^-arene)RuCl(LL)]^+^ (LL = diamino ligand) [84] and [(η^6^-arene)RuCl_2_(pta)] (pta = 1,3,5-triaza-7-phospha-tricyclo-[3.3.1.1]decane) [85], which are typical DNA intercalators. Apart from the research on Ru(II) compounds, there are many exciting developments in the medicinal applications of different metal–arene compounds, especially within the platinum-group metal complexes. Analogous organometallic monochloridoiridium and monochloridorhodium complexes with a general formula [(η^5^-Cp*)MCl(LL’)]^n+^, where Cp* = C_5_Me_5_^−^, n = 0, 1) of the heavier group metals, such as Rh(III) and Ir(III) with neutral or monoanionic chelating aromatic ligands LL’, have been synthesized [86,87]. These compounds have offered similar advantages of high aqueous solubility and inert facial co-ligands that provided a scaffold for the coupling of different functional substituents. Many of the studied compounds have contained polypyridyl ligands (pp), such as 1, 2-naphthoquinone-1-oximate, quinolin-8-ol, etc., Figure 36, which themselves were cytotoxic and have attracted considerable interest as DNA intercalators or groove binders.

Cp*Ir(III) complexes containing the anionic K^2^N,O quinolin-8-olate (qnol^−^) and 1,2-naphthoquinone-1-oximato (npox^−^) ligands have been investigated as potential anticancer agents [86,87]. The IC_50_ value of 0.8 μM for the Ir(III)-Cp* complex towards human melanoma SK-Mel cells was identical to that of its Rh(III)-Cp* counterpart, but somewhat lower activities (IC_50_ = 4.9 and 9.8 μM) were observed towards human melanoma C-32 cells and human gliobastoma SNB-19 cells after a 72 h incubation [87]. Both monochloridoiridium and monochloridorhodium complexes had no impact (IC_50_ > 100 μM) on human melanoma SH-4 cells. During treatments lasting 24 or 48 h, the complexes [(η^5^-Cp*)MCl(npox)] (M = Rh, Ir) inhibited the growth of HeLa and promyelocytic leukemia (HL60) cells. The respective IC_50_ values of 2.2 and 2.0 μM were obtained after 48 h for the monochloridoiridium(III)-Cp* complex [86]. Only a limited 2.3- to 2.5-fold selectivity for the malignant cells was established when compared to normal human umbilical-vein endothelial (HUVEC) cells (IC_50_ = 5.0 μM).

**Figure 37:**
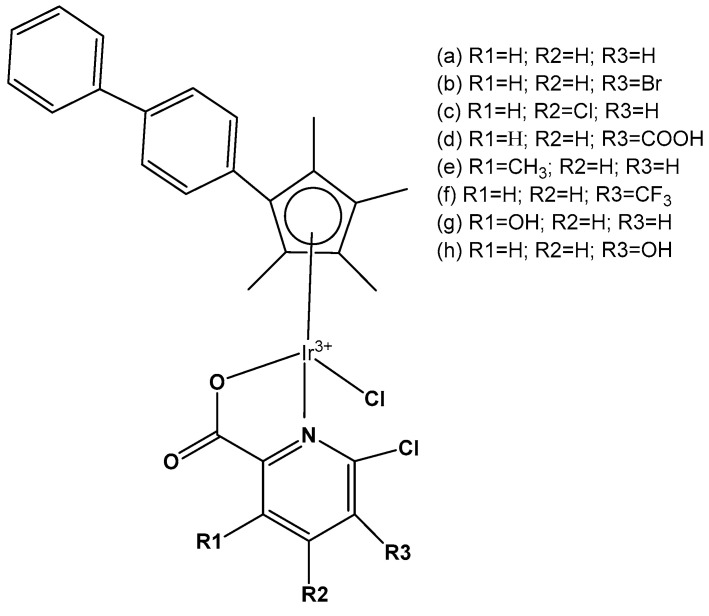
Monochloridoiridium(III) complexes of the general formula [(n5-Cp^xbiph^)Ir(O^N)Cl] of *α*-picolinic acid and its derivatives.

Hao et al. synthesized eight monochloridoiridium complexes, Figure 37(a–h), of the general formula [(*η*^5^-Cp^xbiph^)Ir(O^N)Cl], where Cp^xbiph^ is tetramethyl(biphenyl)cyclopentadienyl, and the O^N is *α*-picolinic acid chelating ligand and its derivatives. Compared with simple cyclopentadiene Cp, its diphenyl derivatives Cp^xbiph^ have shown stronger electron-donor characteristics and could further enhance the rate of ligand exchange and hydrolyze rapidly, therefore improving the overall anticancer activity. The obtained monochloridoiridium complexes have shown more potent antitumor activity against A549 cells by MTT assay [36]. The complex, shown in Figure 37(d), displayed better antitumor activity against the tested lung tumor cells. The complex, presented in Figure 37(f), showed the best results (IC_50_ = 4.41 µM), which was five times greater than that of cisplatin. This complex contained a hydrophobic -CF_3_ group, and this stronger hydrophobicity led to greater cytotoxicity. The complex in Figure 37(f) has shown a larger DNA-binding constant (K_b_) value and binding site number (n) than the complex in Figure 37(a), indicating better anticancer activity of the monochloridoiridium complex, presented in Figure 37(f). These complexes could be transported by serum albumin, bind with DNA, catalyze the oxidation of nicotinamide-adenine dinucleotide (NADH) to NAD^+^ and induce the production of reactive oxygen species, which confirmed their antitumor mechanism of oxidation. The studied monochloridoiridium(III) complexes could enter A549 cells followed by an energy-dependent cellular uptake mechanism, targeting mitochondria and lysosomes, which caused lysosomal destruction, a change in mitochondrial membrane potential, and eventually induced apoptosis.

### 5.4. Monochloridoiridium(III) Cyclopentadienyl Complexes with P,O- and P,P-Donor Chelating Ligands

**Figure 38:**
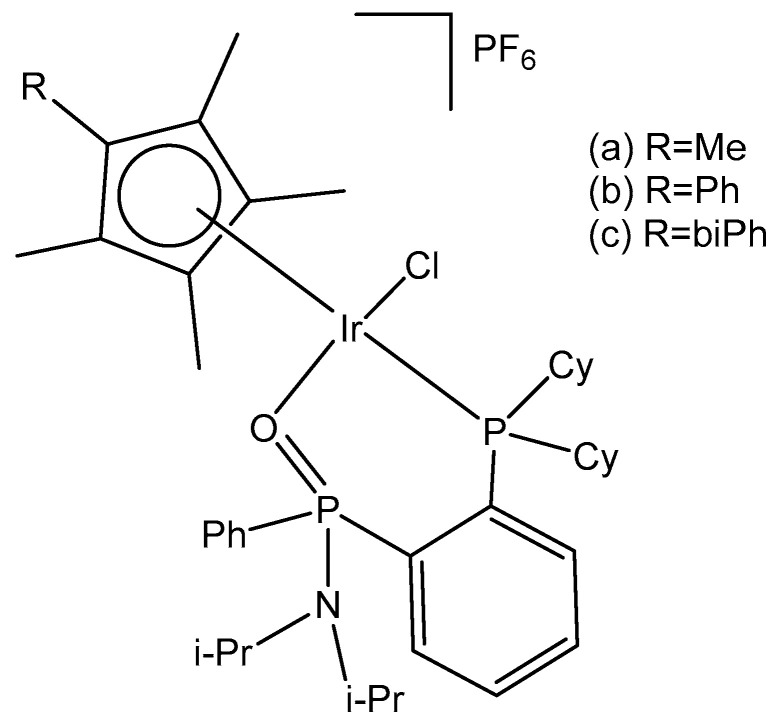
Structure of monochloridoiridium complexes [(Cp*arene)Ir(P^O)Cl]PF_6_ with phosphine phosphonic amide ligand.

Du et al. have synthesized and characterized three monochloridoiridium(III) half-sandwich complexes of the type [(Cp^x^/arene)Ir(P^O)Cl]PF_6_, Figure 38(a–c), where Cp^x^ is pentamethylcyclopentadienyl or its phenyl (Cp^xph^ = C_5_Me_4_C_6_H_5_) or biphenyl (Cp^xbiph^ = C_5_Me_4_C_6_H_4_C_6_H_5_) derivatives with phosphine phosphonic amide ligand, PPOA. The X-ray crystal structures of the complexes, in which the ligands formed six-membered rings with an Ir(III) center, were determined. The authors have studied the antineoplastic activity of the monochloridoiridium complexes by testing them with human cancer cells HeLa and A549 [88]. The obtained results have shown that these complexes exerted remarkable anticancer activities towards HeLa and A549 cells with IC_50_ values of 1–2 µM (for HeLa cells) and 4.4 µM (for A549 cells), which were relatively better than the IC_50_ of the clinically used drug cisplatin. The activity trend was found to be in the order, b > c > a, Figure 38. These results can be attributed to the combinative action of Ir(III) ions and PPOA ligands. The studied monochloridoiridium complexes have exposed potent catalytic activity in the hydrogenation transfer of NADH to NAD^+^, which provided the potential pathway to induce ROS and kill the cancer cells by an oxidation–reduction mode of action. Furthermore, the complex [(η^5^-C_5_Me_5_)Ir(P^O)Cl]PF_6_ arrested the cell cycle at the S and G_2_/M phase. The confocal microscopy study has shown that the most active monochloridoiridium(III) complex damaged lysosomes. Generally, the cell stress, including cell cycle perturbation, the induction of apoptosis, ROS level growth, and the loss of mitochondrial membrane potential, have contributed to the antineoplastic effectiveness of the studied monochloridoiridium(III) complexes (Figure 38) [88].

**Figure 39:**
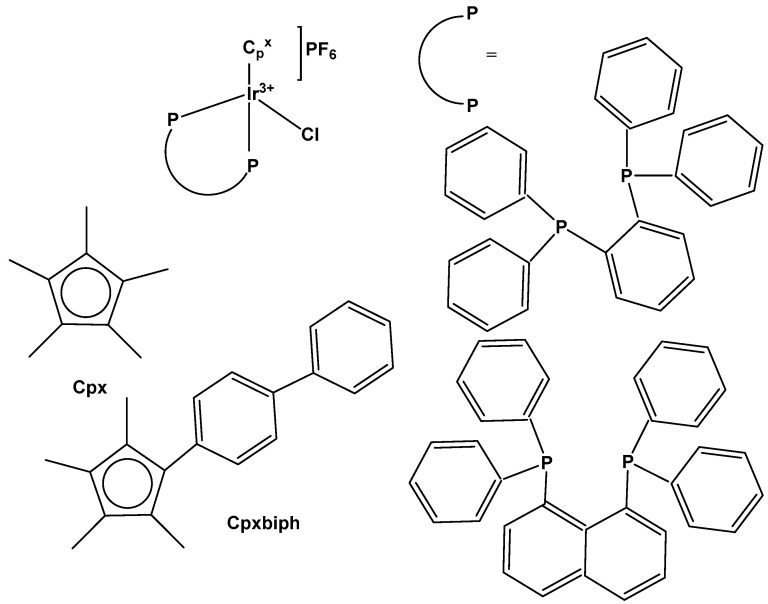
Structures of monochloridoiridium(III) complexes with general formula [(η^5^-Cpx)Ir(P^P)Cl]PF_6_, bearing P^P-ligands 1,2-bis(diphenylphosphino)benzene (dppbz) and 1,8-bis(diphenylphosphino)naphthalene (dppn).

Recently, Li et al. have synthesized four half-sandwich luminescent monochloridoiridium(III) complexes with a general formula [(η^5^-Cp^x^)Ir(P^P)Cl]PF_6_, bearing P^P-chelating ligands 1,2-bis(diphenylphosphino)benzene (dppbz) and 1,8-bis(diphenylphosphino)naphthalene (dppn), Figure 39 [89]. They have been characterized by ^1^H-NMR, ^31^P-NMR spectroscopy, mass spectrometry, elemental analysis and X-ray crystallography. All the monochloridoiridium complexes have displayed better antitumor activity compared to cisplatin against A549 lung cancer cells and HeLa cervical cancer cell lines. The most potent iridium complex with dppn ligand (IC_50_ = 0.502 µM) was 41 times more active than cisplatin towards A549 cells. The complex penetrated into the A549 cancer cells through energy-dependent active transport and was accumulated mainly in lysosomes. It affected the permeabilization of the lysosomal membranes and induced caspase-dependent cell death through lysosomal damage. Apoptosis and autophagy of the cancer cells were observed. No nucleobase adducts of the complex with dppn ligand were detected, which is completely different from the cisplatin mechanism of action. The ROS elevation, reduction in the mitochondrial membrane potential and cell cycle arrest at the G_0_/G_1_ phase have also contributed to the observed cytotoxicity of the monochloridoiridium(III) complex with dppn ligand, presented in Figure 39. This complex also displayed remarkable excited luminescence. The complex with dppbz ligand was nine times more effective in A549 cells and two times more active in HeLa cells than the corresponding [n^5^-Cp*Ir(III)N^N] complexes. The authors have demonstrated that these half-sandwich monochloridoiridium complexes had different anticancer mechanisms of action from that of cisplatin, causing am intracellular ROS outburst and leading to mitochondrial dysfunction, inducing apoptosis and autophagy (Figure 39) [89]. Photophysical studies have shown that all these monochloridoiridium(III) complexes had great potential application in living cells and in vivo imaging due to their notable excited luminescence.

### 5.5. Monochloridoiridium(III) Cyclopentadienyl Dinuclear Complexes

**Figure 40:**
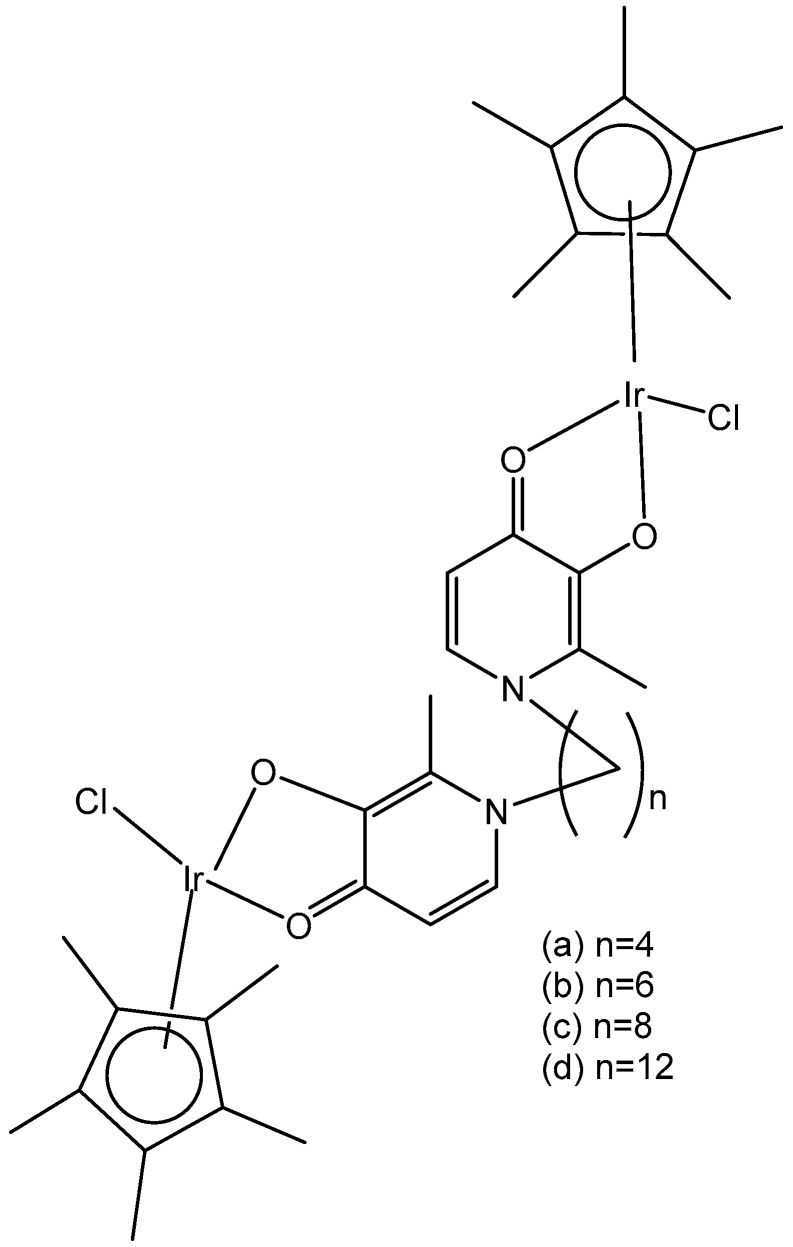
Dinuclear monochloridoiridium(III) complexes.

Parveen et al. have discovered dinuclear monochloridoiridium(III)-Cp* complexes (Figure 40) that had superior in vitro anticancer activity compared to cisplatin [90]. For cytotoxicity evaluation, these complexes were tested against HCT-116, NCI-H460, SW480 and the IC_50_ values of the complex, shown in Figure 40(d), against respective cell lines were 0.38, 0.21, 0.75, 0.88 µM, respectively. The monochloridoiridium(III) complex, Figure 40(d), could induce ROS formation in a concentration-dependent manner. This complex was non-hemolytic up to 125 µM and was less toxic compared to clinically used platinum anticancer drugs. All the dinuclear monochloridoiridium(III)-Cp* complexes (Figure 40) have shown in vitro activity superior to cisplatin and the drug IT-139 while being less toxic in hemolysis and in vivo zebrafish models. The modes of action appeared to be related to DNA damage and ROS-mediated stress pathways.

**Figure 41:**
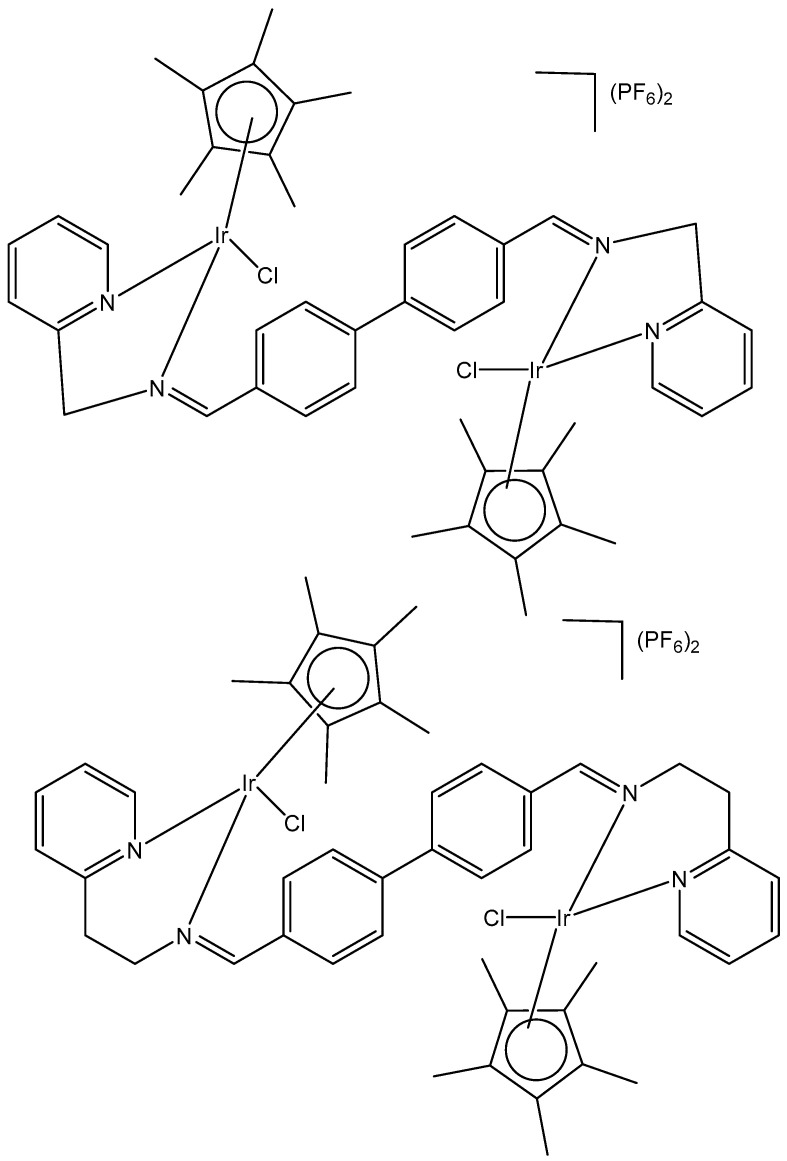
Dinuclear half-sandwich monochloridoiridium(III) complexes [Ir_2_(μ-L^n^)(η^5^-Cp*)_2_Cl_2_](PF_6_)_2_.

A series of dinuclear half-sandwich monochloridoiridium(III) complexes [Ir_2_(μ-L^n^)(η^5^-Cp*)_2_Cl_2_](PF_6_)_2_, based on 4,4′-biphenyl-based bridging Schiff base ligands N,N′-(biphenyl-4,4′-diyldimethylidyne)bis-2-(pyridin-2-yl) methanamine (L^1^) or N,N′-(biphenyl-4,4′-diyldimethylidyne)bis-2-(pyridin-2-yl)ethanamine (L^2^) and Cp* = pentamethylcyclopentadienyl, have been reported [91], Figure 41. The dinuclear monochloridoiridium(III) complexes were characterized by elemental analysis, FT-IR, NMR, ESI-MS. The in vitro cytotoxic activity of the compounds was evaluated at six human cancer (ovarian carcinoma A2780, cisplatin-resistant ovarian carcinoma A2780R, breast carcinoma MCF-7, osteosarcoma HOS, colon carcinoma HT-29 and pancreatic carcinoma PANC-1) cells and at two healthy human cell lines. The complex, containing the L^2^ bridging ligand with a six-membered {MN_2_C_3_} chelate ring, revealed higher cytotoxicity towards A2780 (IC_50_ = 3.1 μM) and MCF-7 (IC_50_ = 6.0 μM) cell lines, as compared with that of the L^1^ bridging ligand, the last forming a five-membered {MN_2_C_2_} chelate ring. The complex with N,N′-(biphenyl-4,4′-diyldimethylidyne)bis-2-(pyridin-2-yl)ethanamine exceeded twice the in vitro cytotoxic activity of cisplatin and showed high selectivity towards the tumor cells (A2780 and MCF-7) over the normal ones, studied in MRC-5 fibroblasts (IC_50_ = 32.3 μM) and the primary culture of human hepatocytes (IC_50_ = 61.3 μM). In contrast to cisplatin, this complex did not induce the cell cycle modification of the treated human ovarian carcinoma A2780 cells. High levels of superoxide anion were induced by this complex at the A2780 cells. The studied dinuclear half-sandwich monochloridoiridium(III) complexes represented the first examples of dinuclear complexes with two {Ir(η-ar)Cl} moieties linked by an N-donor bridging ligand chelating the two metal centers. Recently, this work was followed by less cytotoxic analogous monochloridoiridium(III) complexes containing similar tetradentate N-donor ligands, resulting from 4,4′–methylenedianiline [92] and benzene1,4-diamine [93].

**Figure 42:**
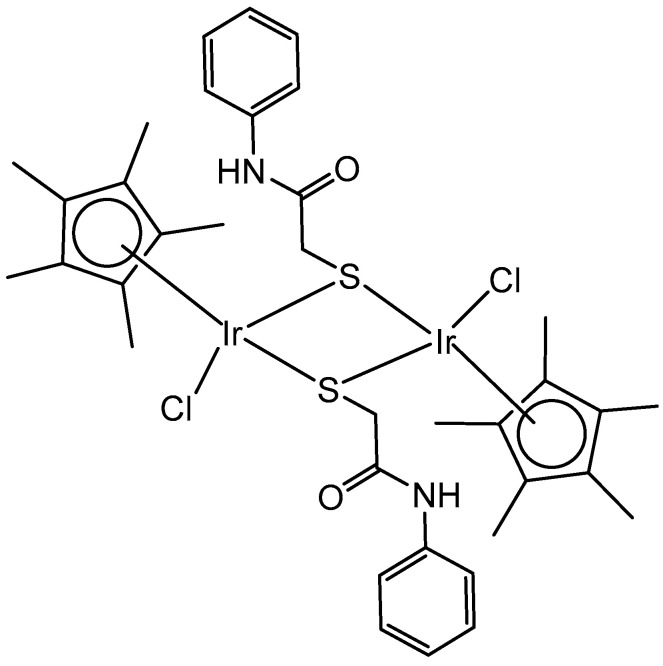
[Ir_2_(μ-mpa)_2_(η^5^-Cp*)_2_Cl_2_] complex of 2-mercapto-N-phenylacetamide (Hmpa).

Kavukcu et al. have reported the synthesis and characterization of a series of mono- and bimetallic Ru(II)– and Ir(III)–arene complexes with different aromatic and aliphatic groups and investigated their effects on Vero and HepG2 cell lines through cell death mechanisms. The electroneutral [Ir_2_(μ-mpa)_2_(η^5^-Cp*)_2_Cl_2_] complex (Figure 42) involves 2-mercapto-N-phenylacetamide (Hmpa), which has a different coordination mode (an S-donor bridging ligand) than its Ru analog [Ru_2_(μ-mpa)_2_(η^6^-pcym)_2_] with mpa coordinated as an N,S-chelating ligand bridging the two metal centers through the sulfur atom of mpa [94]. Both metal complexes exhibited comparable reductions in cancer cell viability expressed in mg mL^−1^, indicating a higher cytotoxicity.

**Figure 43:**
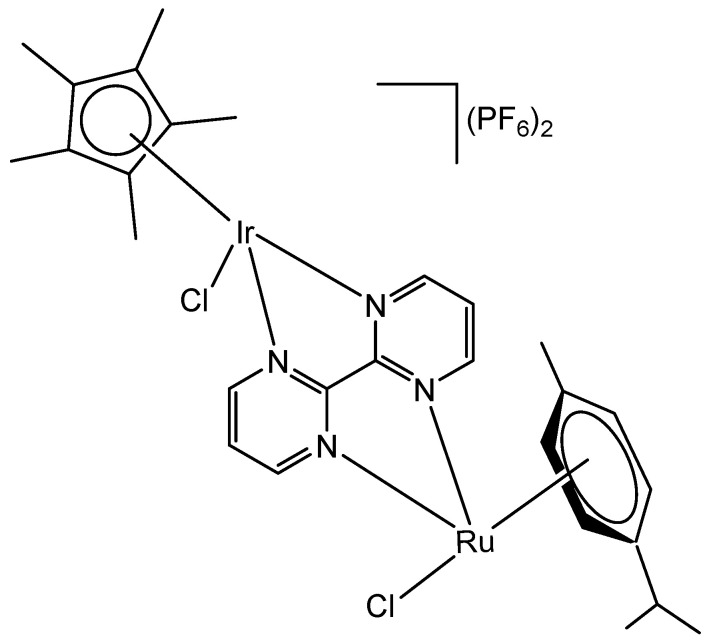
The heterometallic complex [Ir(η^5^-Cp*)Cl(μ-bpm)Ru(η^6^-pcym)Cl](PF_6_)_2_.

Within the framework of development of heterobimetallic complexes, 2,2′-bipyrimidine-based luminescent Ru(II)/Ir(III)–arene monometallic and homo- and hetero-bimetallic complexes have recently been synthesized [95]. The monochlorido iridium–ruthenium complex [Ir(η^5^-Cp*)Cl(μ-bpm)Ru(η^6^-pcym)Cl](PF_6_)_2_ (Figure 43), containing tetradentate 2,2′-bipyrimidine (bpm), has been studied, together with Ir_2_ and Ru_2_ analogs, in MDA-MB-468 (breast carcinoma) and Caco-2 (colon carcinoma) cell lines [95]. Although Ir-Ru was less effective (IC_50_ = 1.9 μM) against MDA-MB-468 cells than Ir_2_ (IC_50_ = 1.8 μM) and Ru_2_ (IC_50_ = 0.9 μM) analogs, this heterometallic compound exceeded (IC_50_ = 6.2 μM) both homometallic analogs (IC_50_ = 32.4 and 46.0 μM for Ir_2_ and Ru_2_, respectively) in Caco-2 cells. Also of interest, this complex was more effective towards Caco-2 cells than both mononuclear parts, i.e., [Ir(η^5^-Cp*)(bpm)Cl]PF_6_ (IC_50_ = 50.4 μM) and [Ru(η^6^-pcym)(bpm)Cl]PF_6_ (IC_50_ = 49.6 μM) (not studied in MDA-MB-468 cells).

**Figure 44:**
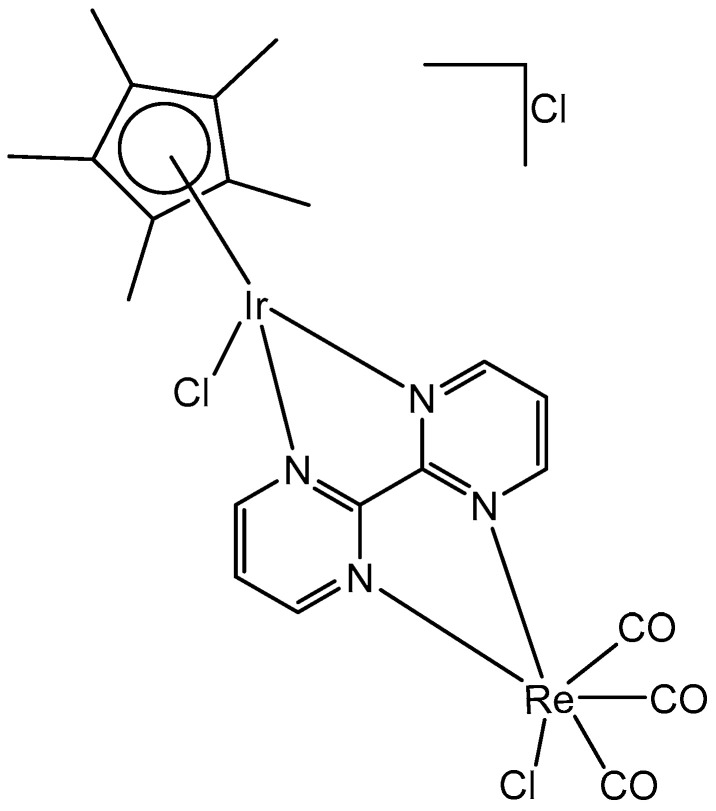
The heterometallic complex [Ir(η^5^-Cp*)Cl(μ-bpm)ReCl(CO)_3_]Cl.

The same group developed a new type of heterometallic monochlorido complex, [Ir(η^5^-Cp*)Cl(μ-bpm)ReCl(CO)_3_]Cl (Figure 44), using the conjugation of the Ir(III)–Cp* and Re(I)-tricarbonyl motif through the same bridging bpm ligand [96]. This complex showed approximately five-fold higher antiproliferative activity against the triple-negative breast cancer MDA-MB-468 cell line (IC_50_ = 24.1 μM) than its Re_2_ homometallic and Ru–Re heterometallic analogs. It was highly selective towards MDA-MB-468 cells in comparison with non-cancerous HaCaT ones (IC_50_ = 234.8 μM). The IrRe complex has been capable of causing apoptosis of the tested cancer cells through inhibition of the G_2_/M phase in the cell cycle in association with a profuse amount of ROS generation and mitochondrial depolarization.

## 6. Conclusions and Prospective

There has been an immense development in exploiting the antiproliferative properties of organometallic chloridoiridium(III) complexes in recent years, which have become a promising alternative to the clinically used antineoplastic drugs. The chloridoiridium(III) complexes with novel modes of action have the capability to broaden the spectrum of cytotoxicity, reduce side effects, and overcome drug resistance. The organometallic chlorido-substituted Ir(III) complexes possess unique attractive properties, including diverse structures, the varied range of ligand substitution rates, potential redox features, higher cellular uptake efficiency, large stokes shifts and lower toxic effects. In most cases, because of the essential differences between the chemotypes of Ir(III) complexes and that of conventional platinum-based drugs, their mechanisms of action are different, which leads to different intracellular targets (mitochondria, lysosomes, etc.), the induction of ROS/RNS generation and eventually the improved spectrum of bioactivity. The mode of action of Ir(III)-based chlorido complexes in cancerous cells has been actively studied, which was highlighted in the current review. In contrast to Pt-based complexes, iridium(III) compounds do not principally target DNA and kill the malignant cells through a different mode of action, which provides much potential and challenges to obtain alternative classes of effective chemotherapeutics and novel biologically active compounds in the near future. In this review, chloridoiridium(III) compounds with promising cytotoxic and antineoplastic activity have been comprehensively overviewed and discussed with regard to their chemical compositions and structures, the nuclearity (mononuclear vs. homo- and hetero- multinuclear complexes), the type of the biologically active ligands with the respective donor sets (C,N-; N,N-; N,O-; N,S- N,P-, etc.), the chelate ring size and other factors. Apparently, various structure–activity relationship correlations can be derived for these complexes reviewed with respect to the above-mentioned factors. The rational approaches to designing chlorido-substituted Ir(III) complexes with improved potency have been analyzed with a focus on the coordination of ligands that themselves are active and that can possibly take part in interactions with specific biotargets or improve the solubility, stability, pharmacokinetics, bioavailability, lipophilicity, cellular accumulation and the intracellular localization of the obtained complexes. Hence, from the reviewed results and findings on numerous chlorido-substituted Ir(III) complexes and their potential efficacy in cancer therapy, it can be concluded that the research and development of novel iridium complexes are a suitable research direction in the field of medicinal chemistry and a viable potential for novel therapeutic approaches and alternatives to conventional anticancer drugs.

## Data Availability

No new data were created or analyzed in this study.
